# Self-Separating Biphasic Electrolyte Enables High-Performance Aqueous Zinc-Ion Batteries via Electron-Enriched Interphase Engineering

**DOI:** 10.1007/s40820-026-02219-3

**Published:** 2026-05-12

**Authors:** Chengwu Yang, Pattaraporn Woottapanit, Qizhi Hou, Zhiqiang Dai, Wanwisa Limphirat, Jiaqian Qin, Xinyu Zhang

**Affiliations:** 1 State Key Laboratory of Metastable Materials Science and Technology, Yanshan University, Qinhuangdao, 066004 People’s Republic of China; 2 Department of Materials Science, Faculty of Science, Chulalongkorn University, Bangkok, 10330 Thailand; 3 International Graduate Program of Nanoscience & Technology (Interdisciplinary), Graduate School, Chulalongkorn University, Bangkok, 10330 Thailand; 4 Synchrotron Light Research Institute (Public Organization), Nakhon Ratchasima, 30000 Thailand; 5 Center of Excellence in Responsive Wearable Materials, Chulalongkorn University, Bangkok, 10330 Thailand; 6 Energy Research Institute, Chulalongkorn University, Bangkok, 10330 Thailand

**Keywords:** Zn-ion batteries, Biphasic electrolyte, PEDOT:PSS, Hydrogen-bonding network, Electrode interphase

## Abstract

Aqueous Zn-ion batteries encounter significant challenges, including unstable electrolyte interfaces and dendrite formation, which curtail their cycle life and metal utilization. Here, we present a self-separating biphasic electrolyte through phase separation of poly (3,4-ethylenedioxythiophene): poly (styrenesulfonic acid) (PEDOT:PSS) conductive polymers in Zn sulfate solution driven by a mechanical shear-ionic crosslinking process. The negatively charged sulfonic groups in PSS disrupt the hydrogen-bonding network of bulk electrolyte and remodel solvation structure of Zn^2+^ ions, enhancing ionic transfer kinetics and facilitating desolvation. Meanwhile, the insoluble PEDOT fibers with continuous conjugated thiophene ring together with sulfonic groups in PSS spontaneously adhere to the electrode surface, establishing a robust electron-rich interphase that regulates electric double layer thickness, promotes uniform Zn deposition and repels sulfate ions. Consequently, this electrolyte allows Zn anodes to achieve exceptional stability and longevity even at larger depths of discharge (68.4% and 94.1%) and delivers outstanding cyclability exceeding 10,000 cycles in Zn||V_2_O_5_ full cells.

**Supplementary Information:**

The online version contains supplementary material available at 10.1007/s40820-026-02219-3.

## Highlights

- A self-separating biphasic electrolyte was developed via mechanical shear-ionic crosslinking phase separation of poly(3,4-ethylenedioxythiophene): poly(styrenesulfonic acid) (PEDOT:PSS).
- The insoluble PEDOT fibers and the soluble PSS establish an electron-rich interphase to improve the Zn anode performance.
- Zn anodes achieve exceptional stability at large depths of discharge and deliver outstanding cyclability exceeding 10,000 cycles in full cells.

**Supplementary Information:**

The online version contains supplementary material available at 10.1007/s40820-026-02219-3.

## Graphical abstract

**Figure:**
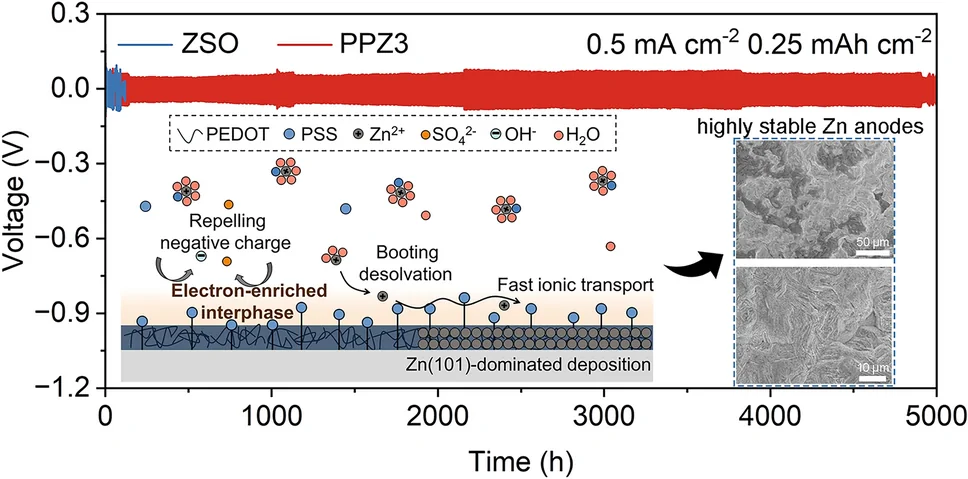

## Introduction

Given the pressing need for a global shift towards renewable energy sources, the development of large-scale, safe and affordable energy storage technologies has become critical [1]. Currently, conventional lithium-ion batteries (LIBs) are limited by the flammable and explosive risks posed by organic electrolytes, as well as the scarcity and high costs of essential metals like lithium and cobalt [2]. As a result, their further advancement is encountering significant obstacles. This situation has spurred considerable interest among researchers in the exploration of next-generation energy storage solutions. Aqueous Zn-ion batteries (AZIBs), which possess intrinsic non-flammability, offer a fundamental solution to safety concerns [3]. Additionally, Zn metal is abundant and cost-effective and has a high theoretical capacity (820 mAh g^−1^/5255 mAh cm^−3^), making it a highly promising alternative technology that is increasingly being investigated [4]. However, the path to commercializing AZIB technology remains fraught with challenges, particularly the uncontrollable growth of Zn dendrites and adverse water-induced side reactions, including the hydrogen evolution reaction (HER), surface corrosion and passivation. These challenges collectively undermine cycle performance, lower Coulomb efficiency and pose risks of separator puncture leading to short circuits. Thus, it is both impressive and vital to effectively navigate the issues related to Zn metal anodes to promote the sustainable development and commercial application of AZIBs.

Numerous efforts have been dedicated to overcoming the aforementioned challenges, including interfacial modification and structural design of Zn metal anodes [5], electrolyte additive engineering [6] and the construction of functional separators [7]. Among these strategies, electrolyte additive engineering, entailing the introduction of inorganic and organic chemicals into aqueous Zn salt solutions, has gained considerable attention as a flexible and efficient approach due to its ease of preparation and high controllability. This method primarily aims to manipulate the hydrogen-bonding network of bulk electrolytes and the ionic solvation structure [8, 9]. Some electrolyte additives with electroactive functional groups can create preferential adsorption or electrostatic shielding layers on the surface of the Zn anode, optimizing the electric double layer (EDL). Actually, the presence of a solid-electrolyte interphase (SEI) is critical for ensuring the stability and longevity of Zn anodes during deep and extended cycling of practical AZIBs. Many functional electrolyte additives, including fluoride and sulfur-containing compounds, have been developed to construct the electrode interphase in situ [10]. However, this rapid and uneven formation often necessitates the self-decomposition of the electrolyte, an irreversible and resource-consuming process. Moreover, with prolonged cycling, the interfacial layer may degrade via dissolution and mechanical stress to cause fracture and the exhausted additive reserves ultimately render the battery’s long-term cycling stability far below expectations. Thus, the pursuit of a versatile electrolyte featuring an optimized ionic solvation structure, a reduced EDL layer and a durable electrode interphase presents a promising avenue for achieving highly stable and high-performance AZIBs.

Poly (3, 4-ethylenedioxythiophene): poly(styrenesulfonic acid) (PEDOT:PSS, PP) is among the most widely used and commercially successful conductive polymer composites, recognized for its outstanding electronic conductivity, mechanical flexibility and chemical/thermal stability, with broad applications in solar cells and flexible electronics [11]. The PEDOT component features a continuous conjugated thiophene ring structure, which allows for extensive electron delocalization across the conjugated system [12]. Conversely, PSS consists of benzene rings and negatively charged sulfonic acid groups, which restrict the delocalization of π electrons on the benzene rings [13]. Within the PP composition, the positively charged insoluble PEDOT chains interact with negatively charged soluble PSS chains through electrostatic forces, causing the previously insoluble PEDOT to be stably dispersed in water. Drawing inspiration from this unique composition and electronic architecture, we developed a self-separating biphasic electrolyte (PPZ) through mechanical shear-ionic crosslinking phase separation of PP polymers in a Zn sulfate (ZnSO_4_, ZSO) solution. The sulfonate groups within the PSS chains tend to crosslink with Zn^2+^ ions in the ZSO solution through robust electrostatic interactions, facilitating the separation of PSS and PEDOT during continuous mechanical stirring. In the PPZ electrolyte, PSS chains remain dissolved, while the insoluble PEDOT settles at the bottom and adheres to the electrode surface. On one hand, sulfonic groups play a crucial role in reorganizing the Zn^2+^ ion solvation structure and hydrogen-bonding network of bulk electrolyte, thereby enhancing the transfer kinetics and desolvation of Zn^2+^ ions. On the other hand, sulfonate groups and the PEDOT adsorbed onto the electrode surface create an electron-rich electrode interphase that regulates the EDL thickness, promotes uniform Zn deposition along the Zn (101) crystal plane and repels SO_4_^2−^ ions to mitigate side reactions. With these comprehensive advantages, the PPZ biphasic electrolyte enables Zn||Zn, Zn||Cu and Zn||V_2_O_5_ cells to achieve much better electrochemical performance. This finding highlights a promising pathway to refine the ionic solvation environment and electrode interphase for fostering greater reliability and efficiency in Zn metal anodes.

## Experimental Section

### Preparation of Self-Separating Biphasic Electrolytes

A 2 M aqueous solution of ZSO was first prepared. PP purchased from Macklin was then added to 5 mL of the 2 M ZSO solution, followed by vigorous stirring for one week at room temperature to ensure complete dispersion. The concentrations of PP in electrolytes were 10, 30, and 50 mM (based on the molar mass of the repeating units of PEDOT and PSS, 325.39 g mol^−1^), respectively, which were denoted as PPZ1, PPZ3, and PPZ5, respectively. The corresponding mass concentrations are 3.25, 9.76 and 16.27 mg mL^−1^, respectively. For comparison, a fully dissolved PEDOT/PSS-ZSO solution (DPPZ) was also prepared. PP was completely dissolved in 5 mL water, a stoichiometric amount of ZSO salt was added to the solution and then the mixture was stirred for one week to ensure homogeneity.

### ***Preparation of V***_***2***_***O***_***5***_*** Cathode Material***

Vanadium pentoxide (V_2_O_5_) was synthesized by heat sintering 10 g of ammonium vanadate (NH_4_VO_3_) in a muffle furnace at 400 °C for 2 h. After cooling to room temperature, the resulting V_2_O_5_ powder was thoroughly ground. Cathode electrodes were fabricated by coating a slurry, comprising V_2_O_5_, conductive carbon, and polyvinylidene fluoride (PVDF) in a 7:2:1 mass ratio, dispersed in N-methyl-2-pyrrolidone (NMP), onto carbon paper. The coated substrates were dried overnight at 75 °C, yielding an average V_2_O_5_ loading of 1 ~ 1.5 mg cm^−2^. For the pouch cells, V_2_O_5_ was pressed onto a piece of stainless steel (SS, 4.8 × 5.6 cm^2^) with the loading mass of ~ 0.4 g.

### Material Characterization

X-ray diffraction (XRD) patterns were collected using a Rigaku D diffractometer to analyze material composition and microstructure. X-ray photoelectron spectroscopy (XPS, Thermo ESCALAB 250XI) probed elemental compositions and chemical environments. Surface morphologies were imaged via scanning electron microscopy (SEM) and confocal laser scanning microscopy (CLSM, Olympus LEXT OLS5000). ^1^H nuclear magnetic resonance (NMR) spectra were recorded on a Bruker 600 MHz spectrometer. Fourier-transform infrared (FTIR) spectra were acquired using a PerkinElmer Spectrum One system. UV–VIS absorption spectra of all samples were recorded by CRAIC 20/30PV. Raman spectra were obtained with a Thermo Fisher DXR spectrometer. In situ micro-computed tomography (micro-CT, Bruker SkyScan 1173) monitored Zn dendrite growth in Zn||Zn symmetric cells under a current density of 1 mA cm^−2^ with a capacity of 1 mAh cm^−2^. Kelvin probe force microscopy (KPFM) was measured by Bruker Dimension Icon.

### Electrochemical Measurements

Zn||Zn symmetric cells, Zn||Cu asymmetric cells, and Zn||V_2_O_5_ full cells were assembled as CR2032 coin cells, using electrodes and Whatman glass fiber separators with diameters of 14 and 19 mm, respectively. To ensure homogeneity and repeatability, the electrolytes were thoroughly stirred and then 150 μL of the electrolytes was added to the cells. Galvanostatic charge–discharge profiles were recorded on a Neware battery testing system. Electrochemical impedance spectroscopy (EIS), Tafel plots, linear sweep voltammetry (LSV), current–time (i—t) curves, and cyclic voltammetry (CV) were performed using a CHI660E electrochemical workstation. The electrochemical stability window of electrolytes was determined via LSV in Zn||stainless steel (SS) asymmetric cells. Ionic conductivities (σ) were calculated using the relationship $\sigma =L/(R \times A)$, where L and A are the separator thickness and area, respectively, and R is the resistance of Zn||Zn symmetric cells measured via EIS over 0.05 Hz to 100 kHz. Zn^2^⁺ transference numbers were derived from i—t curves and EIS data using ${t}_{{Zn}^{2+}}=\frac{{I}_{s}(\Delta V-{I}_{0}{R}_{0})}{{I}_{0}(\Delta V-{I}_{s}{R}_{s})}$, where I₀/R₀ and I_s_/R_s_ are the current/resistance before and after i—t curve measurements, respectively. The electric double layer (EDL) capacitance at the electrolyte-Zn anode interface was calculated from CV curves of symmetric cell using C = εA/d, with ε, A and d representing the electrolyte dielectric constant, electrode area and EDL thickness, respectively. For the cycling tests at the DODs of 68.4% and 94.1%, the Zn thicknesses are 50 and 20 μm, respectively.

### Theoretical Calculations

All ab initio calculations in this work were performed using the Vienna Ab initio Simulation Package (VASP), based on the first-principles density functional theory [14] (DFT). The interactions between ions and valence electrons were described by the projector-augmented wave [15] (PAW) pseudopotential method. The exchange–correlation functional was treated using the Perdew-Burke-Ernzerhof [16] (PBE) generalized gradient approximation. During the calculations, a plane-wave cutoff energy of 500 eV was employed, and the total energy convergence criterion was set to 1 × 10^–6^ eV atom^−1^. Structural relaxation was terminated when the maximum force on any atom was reduced below 0.03 eV Å^−1^. For the binding of PSS with Zn^2+^ and H_2_O, a 3 × 3 × 3 Monkhorst–Pack k-point grid was used. For the structure of PEDOT + PSS adsorbed on a Zn (slab) surface, the Brillouin zone was sampled with a 4 × 4 × 1 Monkhorst–Pack k-point grid. The cell size and atom amount of Zn (slab) surface are listed in Table S1. To calculate the diffusion energy barrier of Zn^2+^ on the Zn (slab) surface, the Climbing Image Nudged Elastic Band [17] (CI-NEB) method was used. The adsorption energy (E_ad_) was employed as a metric to evaluate the affinity of PEDOT + PSS for the Zn(002), (100), and (101) slab surfaces, according to the following equation: E_ad_ = E_Zn-x_—E_Zn_—E_x_, where, E_Zn-x_, E_Zn_ and E_x_ represent the total energy of the Zn slab with the adsorbed PEDOT + PSS molecular cluster, the total energy of the bare Zn slab, and the total energy of the isolated PEDOT + PSS molecule, respectively. A negative E_ad_ value indicates energetically favorable adsorption, and a more negative absolute value of E_ad_ signifies stronger affinity between the PEDOT + PSS molecule and the Zn slab. To assess the stability of the complexes formed by the binding of H_2_O and Zn^2+^ with PSS, the binding energy (E_bind_) was calculated using the following formula: E_bind_ = E_total_—n_x_E_x_—n_y_E_y_, where, E_bind_ represents the binding energy of the formed molecular cluster, E_total_ denotes the total energy of the molecular cluster after relaxation calculations, n_x_ and n_y_ are the number of atoms of each type in the molecular cluster, respectively, E_x_ and E_y_ represent the energy of each atom type in the molecular cluster, respectively.

Molecular dynamics (MD) simulations were performed using the DLPOLY 4.10 software package [18]. We established two distinct simulation models: (1) A system comprising 40 ZnSO_4_ formula units and 1180 water molecules, randomly distributed within the simulation box. (2) A system incorporating 1 PEDOT, 1 PSS anion, 40 ZnSO_4_ formula units and 1180 water molecules, also randomly distributed. In model (2), the polymer was modeled as a charge-neutral PEDOT⁺-PSS⁻ pair. Due to computational limitations, only a minimal representation of the polymer system was considered. Therefore, this model is intended to capture local intermolecular interactions between PP and the electrolyte species, rather than to reproduce the full statistical and structural complexity of bulk PP. The results obtained from this model should thus be interpreted qualitatively. All simulations were conducted within a cubic simulation box of dimensions 50 × 50 × 50 Å^3^. The temperature was precisely controlled at 298 K through the application of a Nose–Hoover thermostat [19]. Periodic boundary conditions and the minimum image convention were implemented to replicate an infinite system. Short-range interactions were treated using a cutoff radius of 12 Å, while long-range electrostatic interactions were computed via the Ewald summation method [20]. Initial configurations were generated by randomly positioning all molecules within the box. System stabilization involved an initial equilibration phase under the NPT ensemble for approximately 2 ns, utilizing a time step of 1 fs. Subsequently, production simulations were performed under the NVT ensemble for a duration of 20 ns. Analysis of structural properties, specifically radial distribution functions (RDFs), was based on trajectories extracted from the final 10 ns of the production runs. Intermolecular interactions were modeled as follows: The SPC/E water model was employed for water-water interactions [21], incorporating constraints on bond lengths and angles [22]. For all other atom types, including PEDOT, PSS, Zn^2+^ and SO_4_^2−^, the DREIDING force field was utilized [23]. Lennard–Jones (LJ) parameters (${\sigma}_{ij}$, ${\varepsilon}_{ij}$) for atom pair interactions were determined from individual atomic parameters (${\sigma}_{ii}$, ${\varepsilon}_{jj}$) using the Lorentz-Berthelot mixing rules as follows [24]: ${\sigma}_{ij}=\frac{{\sigma}_{ii}+{\sigma}_{jj}}{2}; {\varepsilon}_{ij}=\sqrt{{\varepsilon}_{ii}{\varepsilon}_{jj}}$. Partial atomic charges for PEDOT, PSS and SO_4_^2−^ were derived from quantum chemical calculations conducted with the ORCA program. These charges were obtained at the HF/6—31G(d) level of theory via electrostatic potential (ESP) fitting, employing the CHELPG method [25]. The Zn^2+^ ion was assigned a formal charge of + 2, while water molecules were described using the standard SPC/E charge model.

## Results and Discussion

### Electrolyte Design Validation and Structures

**Fig. 1:**
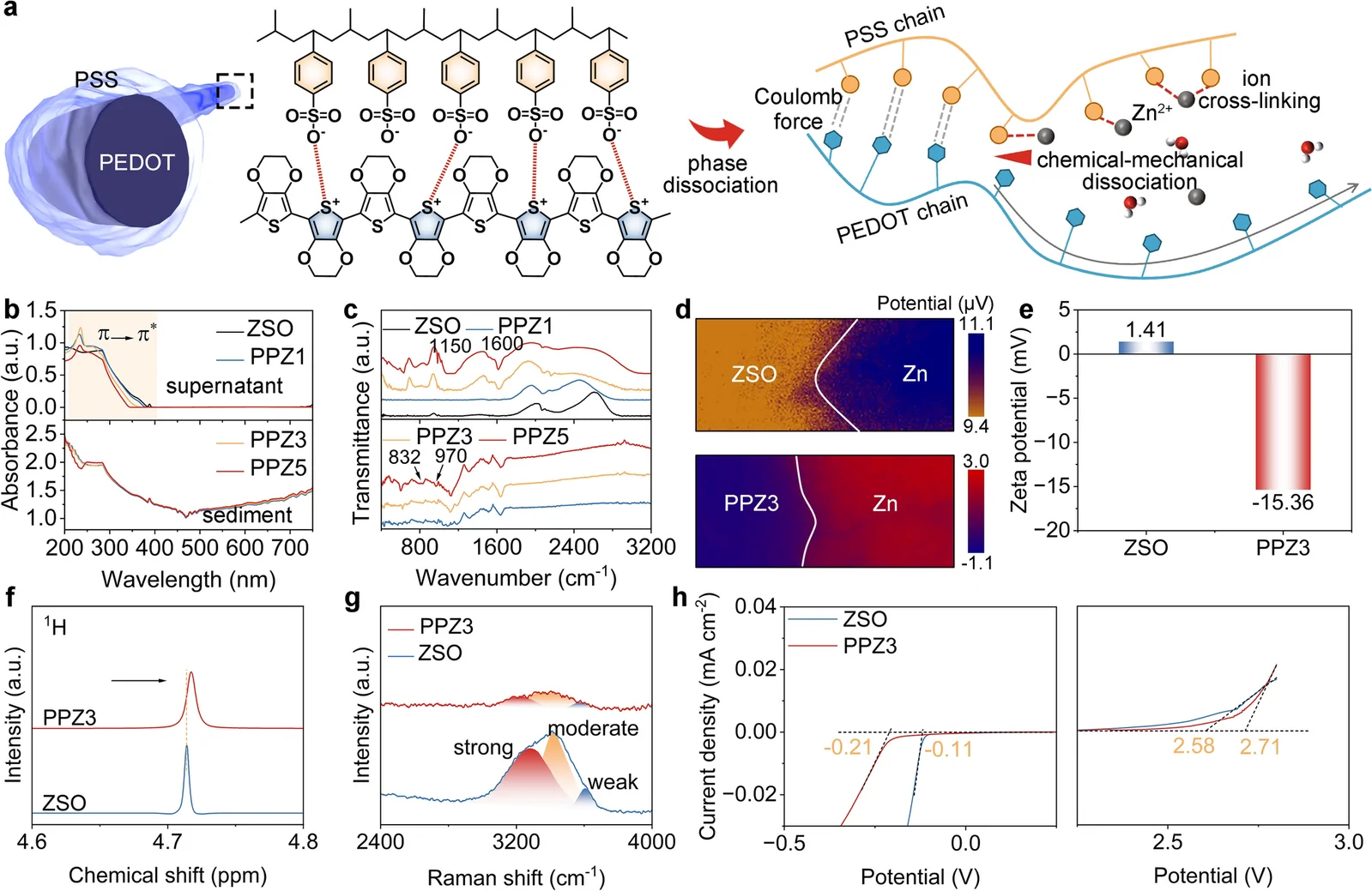
**a** Schematic illustrations of DPPZ and PPZ solutions before and after sedimentation with corresponding digital photos. **b** Mechanism of phase dissociation of PEDOT and PSS chains. **c** UV–VIS absorption and **d** FTIR spectra of PSS supernatant and PEDOT sediment of the PPZ. **e** Potential distribution of ZSO and PPZ3 on Zn electrode surfaces. **f** Zeta potential of ZSO and PPZ3. **g** 1H NMR and Raman spectra of ZSO and PPZ3. **h** ESW of ZSO and PPZ3 tested in Zn||stainless steel cells

PP exhibits inherent water solubility arising from the negatively charged sulfonic groups in PSS chains (Fig. S1). Incorporation of ZSO salt into aqueous PP solutions (designated DPPZ) preserves the original appearance and structure of PP in water (Fig. S2a). Notably, adding PP to ZSO solutions could compromise its water solubility, leaving undissolved fibers dispersed even after one week of vigorous stirring (Fig. S2b). After 24 h of sedimentation, the DPPZ solution separates into a distinct two-phase system featuring a colorless transparent supernatant and a blue subnatant (Fig. S3). For PPZ samples, undissolved fibers settle spontaneously at the bottom of the solution, while the supernatant acquires a blue hue. This self-separation behavior of PP in ZSO stems from phase dissociation between soluble PSS and hydrophobic PEDOT (Fig. 1a). Scanning electron microscopy (SEM) images and Energy-dispersive X-ray spectroscopy (EDS) mapping of exfoliated PEDOT fibers are provided in Fig. S4. Actually, in the PP polymer, PSS and PEDOT are electrostatically cross-linked via Coulomb interactions between sulfonic groups in PSS chains and thiophene rings in PEDOT. Density functional theory (DFT) calculations reveal sulfonate groups have a lower binding energy with Zn^2+^ ions (-5.10 eV) than with H_2_O (− 4.78 eV), indicating the preferential interaction with Zn^2+^ in ZSO solution (Fig. S5). X-ray photoelectron spectroscopy (XPS) confirms electrostatic interactions between Zn^2+^ and sulfonate groups (Fig. S6), which lower the binding energy of Zn 2*p* orbitals (ZSO: 1022.9 eV of Zn 2*p*_3/2_ and 1046.1 eV of Zn 2*p*_1/2_, PPZ: 1022.7 eV of Zn 2*p*_3/2_, 1045.8 eV of Zn 2*p*_1/2_) due to the electron donor from the sulfonate group to Zn^2+^. As a result, these strong Zn^2+^-sulfonate interactions, combined with mechanical shear during stirring, shield the original cross-linking between sulfonate and thiophene, thereby inducing dissociation of PEDOT and PSS.

Ultraviolet–visible (UV–Vis) absorption spectra were employed to confirm the dissociation of PEDOT and PSS in PPZ samples. PEDOT sediment was isolated via centrifugation and thoroughly washed with deionized water. As shown in Fig. 1b, the PSS supernatant displays a distinct absorption edge in the UV range (350—400 nm) and an intense peak at ~ 235 nm, which ascribes to the electron transition from the low-energy π orbital (ground state) to high-energy π* orbital (excited state) in PSS benzene rings upon UV photon absorption [26, 27]. The finite and localized conjugation of benzene rings precludes visible light absorption. In contrast, PEDOT sediment exhibits full absorption across the UV to infrared range (200–750 nm), resulting from its long and continuous conjugated chain system that enables delocalized electron migration even with low-energy infrared photon absorption. This striking difference in light absorption confirms the efficacy of the mechanical-chemical dissociation strategy for PP polymers. For DPPZ solutions, UV–VIS spectra indicate no separation of PEDOT and PSS (Fig. S7). Fourier transform infrared (FTIR) spectra further support these findings that the PSS supernatant shows characteristic peaks for S=O symmetric stretching (~ 1150 cm^−1^) and C=C symmetric stretching (~ 1600 cm^−1^) (Fig. 1c) [28], while PEDOT sediment exhibits stretching vibrations of thiophene rings at ~ 832 cm^−1^ and C-S-C stretching vibrations at ~ 970 cm^−1^ [29, 30]. On account of the self-separating biphasic electrolyte system, it can be hypothesized that the PPZ electrolyte is capable of spontaneously forming an electron-enriched polymeric interphase on electrode surfaces and meanwhile regulating Zn^2+^ solvation structure in the bulk electrolyte, accelerating Zn^2+^ transfer kinetics.

Kelvin probe force microscopy (KPFM) was used to analyze electrolyte surface potentials on Zn anodes. PPZ solution containing 0.03 mM PP concentration (PPZ3) is selected for subsequent detailed investigations. The ZSO electrolyte exhibits a positive surface potential on Zn (Fig. 1d), indicative of a high work function and low interfacial energy barrier. This means that ZSO possesses high activity toward Zn^2^⁺ ions and water molecules, which can accelerate uneven Zn^2+^ reduction and dendrite formation at anode tips while promoting water-induced side reactions. For the PPZ3 biphasic electrolyte, sulfonate groups in PSS chains and the conjugated electron structure of PEDOT induce a significant reduction in surface potential to negative values and construct an electron-rich interphase, corresponding to a higher interfacial energy barrier that facilitates uniform Zn^2+^ nucleation and deposition and suppresses side reactions. Moreover, Zeta potential measurements reveal a value of -15.36 mV for PPZ3 (Fig. 1e), more negative than that of ZSO (1.41 mV). This reduction enhances Zn^2+^ accessibility to the electrode surface for nucleation while repelling SO_4_^2−^ anions. Nuclear magnetic resonance (NMR) spectroscopy probed ^1^H chemical environments to investigate PPZ3 effects on water molecules (Figs. 1f and S8). Compared to ZSO, ^1^H nuclei resonaces of the biphasic electrolytes show clear chemical shifts. The upfield shift arises from newly formed hydrogen bonds between dissolved PSS chains and water molecules that increase electron density around H nuclei [31, 32]. FTIR spectra further corroborate additional intermolecular hydrogen bond formation in the PPZ3 bulk electrolyte (Fig. S9). Raman spectra then quantified hydrogen-bonding network evolution. O–H stretching vibrations at 3000–3800 cm^−1^ were deconvoluted into three peaks corresponding to strong, moderate and weak hydrogen-bonding interactions (Fig. 1g). All three are significantly weaker in PPZ3 than in ZSO, indicating PSS and PEDOT polymers disrupt the original water-water hydrogen-bonding network. The increased pH values of electrolytes from 4.50 of ZSO to 5.68 of PPZ3 further proves this deduction. This restructuring helps reduce free water molecules and suppress undesirable interfacial side reactions. LSV measurements directly affirm that PPZ3 effectively passivates Zn electrodes against HER activity (Fig. S10). Furthermore, the reconfigured hydrogen-bonding network enables PPZ3 to achieve a wider electrochemical stability window (ESW) of 2.92 V (Fig. 1h), compared to 2.69 V for pure ZSO.

### Electrochemical Properties of Electrolytes

**Fig. 2:**
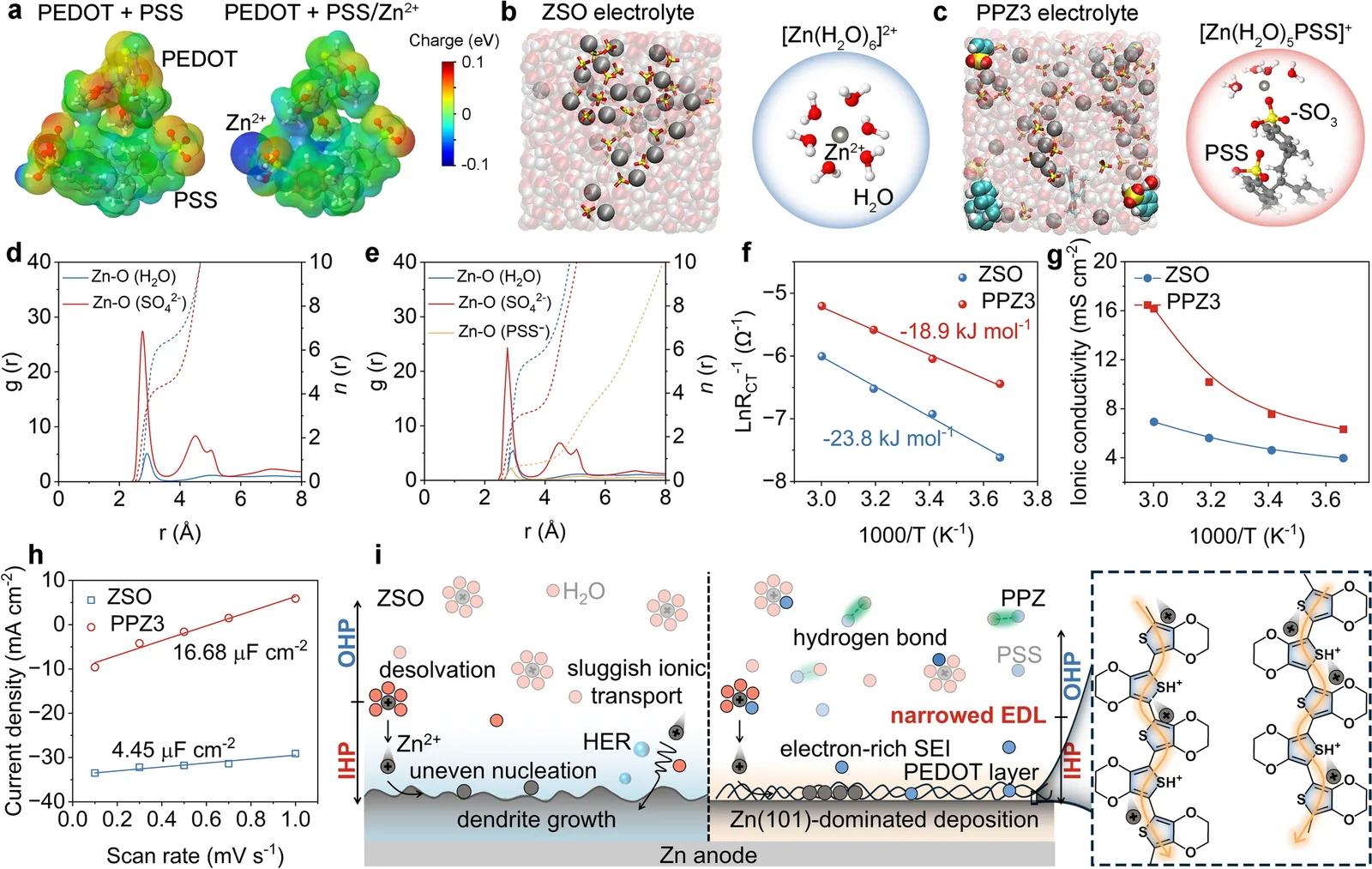
**a** ESP of PEDOT + PSS and the units of PEDOT + PSS after combining with one Zn^2+^ ion. MD snapshots with the corresponding solvation structure of the Zn^2+^ ion in **b** the ZSO and **c** PPZ3 electrolytes. The RDFs of Zn^2+^-O in **d** the ZSO and **e** PPZ3 electrolytes. **f** The calculated activation energy (E_a_) of Zn^2+^ ions in the electrolytes. **g** Ionic conductivity and **h** EDL capacitance of ZSO and PPZ3 electrolytes. **i** The mechanism of Zn^2+^ transport and deposition in the ZSO and PPZ3

Electrostatic potential (ESP) calculations were conducted to precisely map the binding sites of Zn^2+^ ions, a critical step in elucidating the molecular basis of their interaction with PEDOT and PSS. A fundamental principle governing such interactions is that atomic sites or functional groups with more negative ESP values exhibit enhanced reactivity toward electrophilic species, thereby rendering them thermodynamically favored targets for the coordination and nucleophilic attack by Zn^2^⁺ ions. As illustrated in Figs. 2a and S11, the sulfonic group localized at the terminal regions of PSS chains displays the most negative isosurface charge density within the system, in stark contrast to the uniformly positive ESP profile observed across the entire PEDOT molecular fragment. This pronounced discrepancy in ESP distribution not only attests to the strong intrinsic electronegativity of the sulfonic groups but also underscores their high chemical affinity for Zn^2+^ ions via electrophilic addition reactions. Indeed, the introduction of Zn^2+^ ions into the PEDOT + PSS composite system results in the specific coordination of Zn^2+^ with the terminal sulfonic groups of PSS chains, accompanied by a substantial redistribution of electron density within the coordination sphere. This preferential binding of Zn^2+^ to PSS chains further corroborates the role of Zn^2+^ ions in facilitating the dissociation of PP molecules. Given the intimate link between metal ion coordination and solvation structure, we hypothesized that the PSS-Zn^2+^ interaction would alter the solvation environment of Zn^2+^ ions. To test this hypothesis, we conducted comparative molecular dynamics (MD) simulations of Zn^2+^ solvation in the electrolytes. For the ZSO electrolyte (Fig. 2b), Zn^2+^ ions are fully hydrated to form the archetypal [Zn(H_2_O)_6_]^2+^ octahedral solvation complex that is known to induce sluggish desolvation kinetics at electrode interfaces and promote parasitic surface side reactions. As expected, the sulfonic group intercalates into the primary solvation shell of Zn^2+^ (Fig. 2c), replacing one coordinated water molecule to form a new [Zn(H_2_O)_5_PSS]^+^ coordination complex. Analysis of radial distribution functions (RDFs) further quantifies this structural transition, in which Zn^2+^ exhibits a coordination number (CN) of 6.4 with H_2_O molecules and 4.4 with SO_4_^2−^ anions in the ZSO electrolyte (Fig. 2d), while these CN values of the PPZ3 electrolyte are reduced to 5.6 and 3.1, respectively (Fig. 2e). These data provide direct evidence that the PSS chains modulate the Zn^2+^ solvation shell by displacing water molecule and weakening the interaction between Zn^2+^ and SO_4_^2−^ anions.

The Zn desolvation barrier in the electrolytes was then assessed to quantify the ionic activation energy (E_a_). The tested EIS plots with the corresponding equivalent circuit are shown in Fig. S12. Figure 2f demonstrate that compared with 23.8 kJ mol^−1^ in the ZSO electrolyte, the Zn^2+^ ions in the PPZ3 electrolyte exhibit a reduced E_a_ of 18.9 kJ mol^−1^, consistent with a lower Zn^2+^ desolvation barrier and enhanced reaction kinetics imparted by PPZ3. Moreover, the dissolution of PSS optimizes both the ionic solvation structure and desolvation dynamics, endowing the PPZ3 electrolyte with higher ionic conductivity than ZSO across various temperatures (Figs. 2g and S13). EIS plots recorded before and after i—t measurements (Fig. S14) further confirm that the PPZ3 electrolyte accelerates ionic transport, with a Zn^2+^ transference number (${t}_{{Zn}^{2+}}$) of 0.88 that surpasses the 0.56 value of ZSO. Thence, these above results demonstrate that the PPZ3 biphasic electrolyte, comprising soluble PSS chains with abundant sulfonic acid moieties and insoluble PEDOT with a continuous conjugated framework, can effectively modulate and facilitate both Zn^2+^ desolvation and ion transport. To probe the evolution of EDL capacitance and thickness, cyclic voltammetry (CV) measurements were carried out on Zn symmetric cells with different electrolytes via utilizing the relationship of C = εA/d [33] (where ε, A and d represent the electrolyte dielectric constant, electrode area and EDL thickness, respectively). As shown in Figs. 2h and S15, PPZ3 electrolyte engenders an EDL capacity of 16.68 μF cm^−2^, approximately 4 times higher than that of ZSO (4.45 μF cm^−2^). Such marked capacitance enhancement unveils that the unique composition and architecture of PPZ3 electrolyte can profoundly alter the EDL structure and reduce its thickness (Fig. 2i), which in turn reinforces rapid ionic transport and enables subsequent uniform Zn nucleation and dense deposition.

### Effects of Electrolytes on Zn Plating and Stripping

**Fig. 3:**
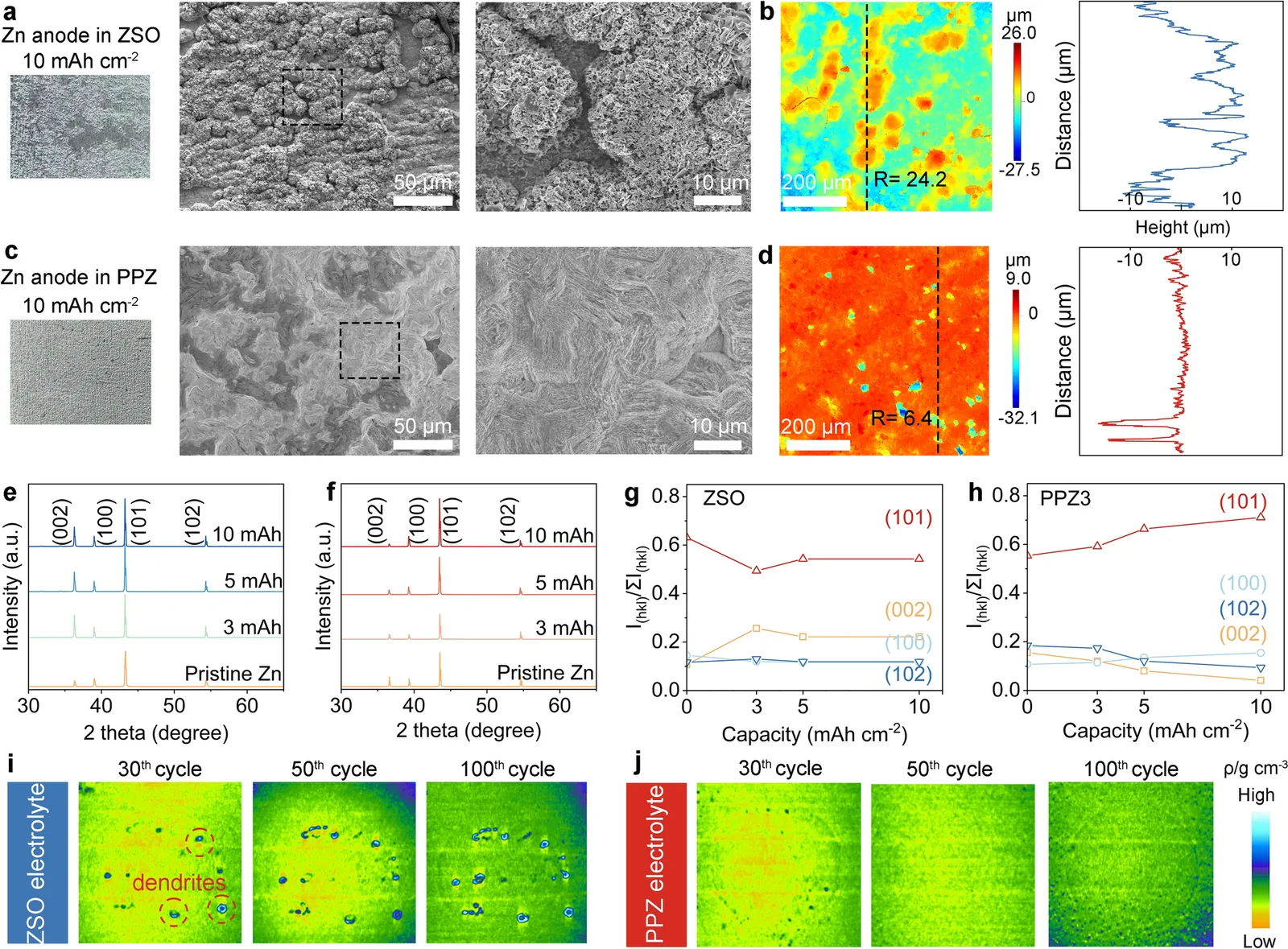
Microstructure of Zn anodes after Zn^2+^ deposition by SEM and CLSM images in **a, b** ZSO and **c, d** PPZ3 electrolytes. XRD patterns of Zn electrodes with **e** the ZSO and **f** PPZ3 after Zn deposition. Relative texture coefficients of Zn planes of **g** ZSO and **h** PPZ3 electrolytes. In-situ micro-CT experiments of Zn cells with **i** ZSO and **j** PPZ3 electrolytes

To visually assess the influence of the PPZ on the ionic deposition process, we examined the microstructure and surface morphology of Zn electrodes using SEM. After depositing a Zn capacity of 10 mAh cm^−2^ in ZSO electrolyte, moss-like Zn dendrites interspersed with Zn nanosheets are observed across the electrode surface (Figs. 3a and S16a). Confocal laser scanning microscopy (CLSM) further reveals scattered protrusions with a high roughness value (R) of 24.2 (Figs. 3b and S17a). By contrast, the Zn deposited in the PPZ3 forms a densely stacked layer that covers the electrode uniformly (Figs. 3c and S16b). CLSM image confirms a smooth and homogeneous surface with a significantly lower roughness of 6.4 (Figs. 3d and S17b). These results demonstrate that the PPZ3 can promote flat and dendrite-free Zn deposition. XRD patterns of Zn electrodes under various deposition capacities (3, 5 and 10 mAh cm^−2^) were used to analyze deposition behavior. For ZSO electrolyte, characteristic peaks of metallic Zn (JCPDS 04—0831) are observed at 36.3°, 39.0°, 43.2°, and 54.3°, corresponding to the (002), (100), (101), and (102) planes, respectively, under all deposition conditions (Fig. 3e). However, in PPZ3, the peak intensities of the (002), (100), and (102) planes decrease with increasing deposition capacity, while the (101) peak intensifies and becomes dominant at 10 mAh cm^−2^ (Fig. 3f). Relative texture coefficients (I_(hkl)_/Ʃ_(hkl)_), where I_(hkl)_ denotes the XRD peak intensity of each plane and Ʃ_(hkl)_ represents the total intensity of all planes, were calculated to evaluate preferential deposition orientation and plane selectivity. These coefficients in ZSO electrolyte remain nearly constant beyond 5 mAh cm^−2^ (Fig. 3g), indicating disordered growth and a lack of plane orientation, consistent with dendritic formation. In PPZ3 electrolyte, the (101) texture coefficient rises markedly to 71% at 10 mAh cm^−2^, while values for the (002) and (102) planes drop considerably (Fig. 3h). This trend confirms that PPZ3 facilitates primary Zn^2+^ deposition along the (101) plane, aligning with the uniform morphology observed by SEM. To exclude substrate interference, XRD patterns of Zn deposited on titanium (Ti) foil were also conducted (Fig. S18), which exhibits similar trend with on Zn substrate. In-situ micro-computed tomography (micro-CT) was performed on Zn||Zn symmetric cells to track morphological changes over multiple cycles. Materials with different densities are distinguished by color variation in micro-CT images. As anticipated, ZSO electrolyte exhibits discontinuous color variations at the 30th cycle (Fig. 3i), reflecting dispersed dendrites and a rough surface morphology that deteriorates progressively in subsequent cycles. In contrast, PPZ3 electrolyte maintains a smooth cycled surface with uniform color distribution throughout cycling (Fig. 3j), due to the uniform electric field flux afforded by PEDOT fibers and smooth and dense Zn deposition along the (101) plane.

### Zn Deposition Mechanism of Electrolytes

**Fig. 4:**
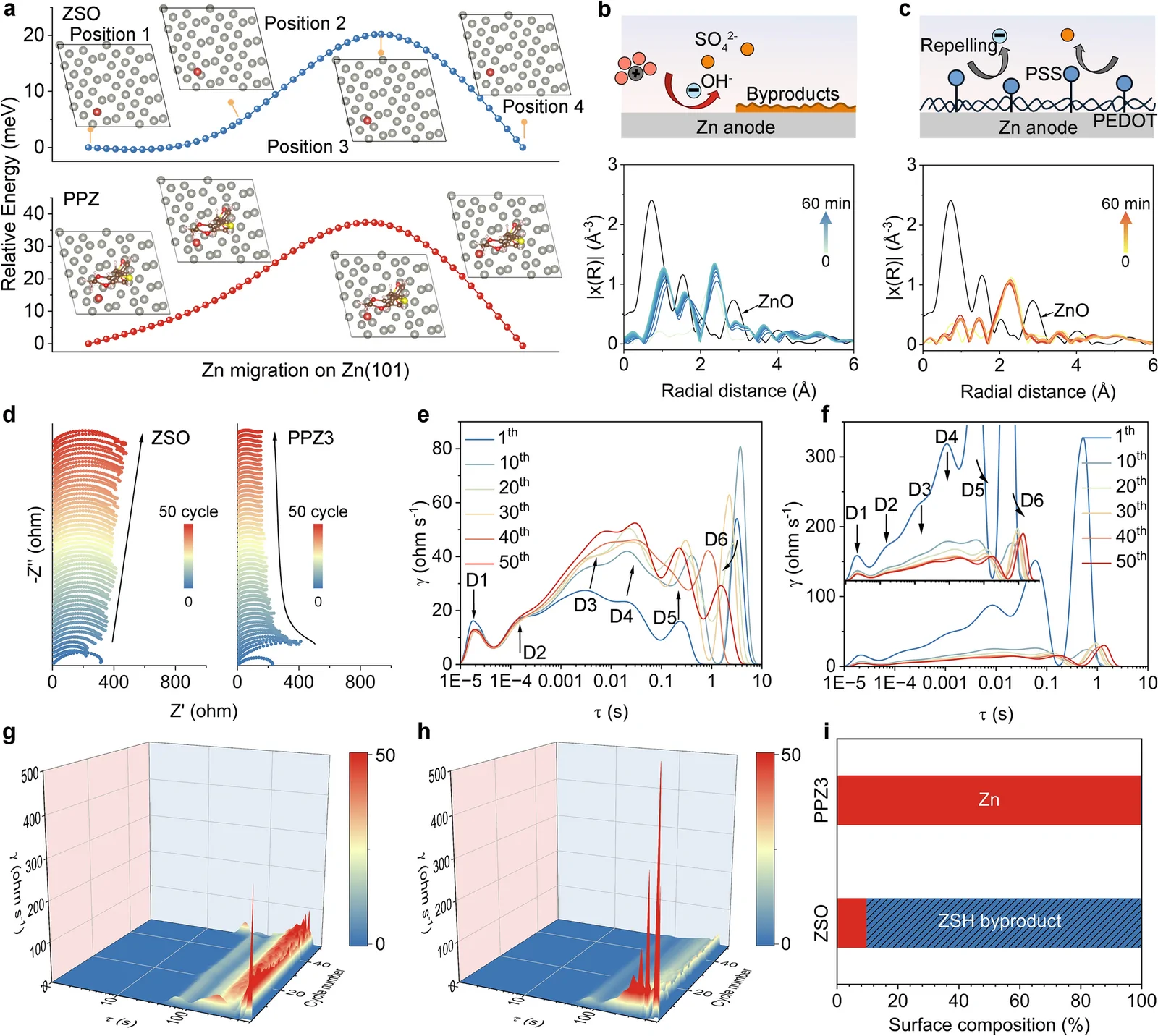
**a** Simulations of Zn^2+^ migration on the Zn surface. R-space data of Zn K-edge of Zn anodes with **b** ZSO and **c** PPZ3 electrolytes tested by in-situ XAS spectra with the schematic illustration of byproduct formation. **d** In-situ EIS tests of symmetric cells with different electrolytes. DRT results of Zn symmetric cells with **e, g** ZSO and **f, h** PPZ3 electrolytes. **i** Surface composition of Zn electrodes after cycling

To gain further insight into Zn^2+^ deposition behavior and mechanism in PPZ3, DFT calculations were conducted to evaluate interactions between PEDOT + PSS and the Zn substrate. PEDOT + PSS exhibits a lower adsorption energy of -1.01 eV on the Zn(101) plane (Fig. S19), indicating preferential adsorption on this facet [34]. Additionally, their interface exhibits localized charge distribution and strong electronic interactions that facilitate rapid Zn^2+^ reduction to Zn^0^ and uniform ionic nucleation. I-t curves confirm this behavior, which, unlike the continuously rising current in ZSO, PPZ3 yields a stable current after 50 s (Fig. S20), signaling a fast transition from 2 to 3D diffusion that favors compact Zn deposition. Zn^2+^ migration on the Zn substrate was simulated to further elucidate electrolyte effects (Figs. 4a and S21). With the synergistic effect of PEDOT and PSS, migration energies of Zn^2+^ at all diffusion positions are significantly higher than those in the ZSO electrolyte. After water adsorption on the Zn surface, Zn^2+^ migration also show same trends (Fig. S22). This elevated ion migration energy barrier indicates that PEDOT and PSS adsorbed on Zn (101) planes retard Zn^2+^ movement and prevent subsequent ion aggregation, which in turn slows nucleation and growth along the Zn[101] direction, as a result preserving the Zn(101) plane. This accords with the observation in SEM images. Thus, in contrast to the rampant dendrite formation in ZSO, PPZ3 electrolyte supports a stable Zn anode through a multifunctional collaborative mechanism (Fig. 2i). First, the dissolved PSS polymer coordinates Zn^2+^ to modify the solvation structure and lower the desolvation barrier. Second, the insoluble PEDOT polymer fibers adhering to the Zn surface facilitate rapid ionic transport and reduction, leveraging the delocalized conjugation π-π bonds with excess electrons in the long and continuous polymer chains. Simultaneously, PEDOT fibers and sulfonate groups adsorbed on the Zn surface form an electron-rich electrode interphase that blocks SO_4_^2−^ and water-decomposited OH^−^ via electrostatic repulsion and steric hindrance, eventually suppressing surface corrosion and byproduct formation [35]. This dual mechanism is also the main reason for the improvement of electrolyte stability. Tafel curves as a well-established electrochemical technique for evaluating corrosion kinetics show that the PPZ3 electrolyte exhibits a substantially lower corrosion current density (i_corr_) of 1.36 mA cm^−2^ (Fig. S23), in sharp contrast to the 2.69 mA cm^−2^ measured for the ZSO electrolyte. This verifies the robust anti-corrosion capability of the PPZ3 electrolyte.

In-situ X-ray absorption spectra (XAS) were tested on Zn||Zn cells to analyze the formation and accumulation of byproducts. As ion deposition proceeds at 10 mA cm^−2^ (Figs. 4b and S24a), the r-space signals of Zn K-edge in ZSO electrolyte progressively resemble those of ZnO. This resemblance implies a transition in the chemical coordination environment of Zn atoms towards a state similar to that in ZnO. On the contrary, Zn anode with PPZ3 exhibits no evident shifts or transformations in Zn K-edge (Figs. 4c and S24b), elucidating that the PPZ biphasic electrolyte can effectively interdict byproduct formation. This function is also validated by in-situ EIS measurements of Zn||Zn cells. The PPZ3 cell yields a lower initial resistance than ZSO (Fig. 4d). With cycling, the resistance in PPZ3 gradually decreases and stabilizes, whereas it increases markedly in ZSO. Distribution of relaxation times (DRT) analysis derived from EIS plots was used to dissect the subtle kinetic variations in interfacial charge transport. As depicted in Fig. 4e, f, the DRT curves of the ZSO and PPZ3 electrolytes present six distinct peaks [36, 37]. Specifically, peak D1 corresponds to electronic transport within the anode. Peak D2 is associated with the adsorption and desolvation of Zn solvation structures. Peaks D3 and D4 are attributed to the migration and reduction of Zn^2+^ ions at the Zn electrode surface. Peak D5 denotes the interfacial charge-transfer process of Zn^2+^ and peak D6 represents the diffusion process of Zn^2+^ within the bulk electrolyte. Notably, upon prolonged cycling, the peak intensities of D2–D5 in the ZSO electrolyte are significantly elevated. This observation indicates that the formation and accumulation of parasitic byproducts give rise to exacerbated kinetic barriers for Zn^2+^ migration and reduction. In stark contrast, the PPZ3 electrolyte exhibits a pronounced reduction in the intensity of all DRT peaks, which implies that the electron-rich electrode interphase sustains rapid kinetics for Zn^2+^ migration and reduction. Furthermore, the 3D DRT contour maps presented in Fig. 4g, h directly manifest this disparity, demonstrating that the PPZ3 electrolyte preserves a stable electrode–electrolyte interface with increasing cycle numbers. XRD patterns were utilized to quantitatively detect the surface composition of cycled Zn anodes (Fig. S25). ZSO electrolyte induces extensive accumulation of Zn_4_SO_4_(OH)_6_·5H_2_O (ZSH) with a high composition ratio of 90.3% (Fig. 4g). Distinctly, the PPZ3 electrolyte, through efficient SO_4_^2−^ and OH^−^ repulsion by sulfonic groups and PEDOT polymer fibers, greatly reduces the ZSH byproduct ratio on the Zn surface. The damped XPS signal of S 2*p* recorded in the PPZ3 also confirms the effect of sulfonic groups and PEDOT polymer fibers (Fig. S26).

### Electrochemical Performance of Symmetric and Asymmetric Cells

**Fig. 5:**
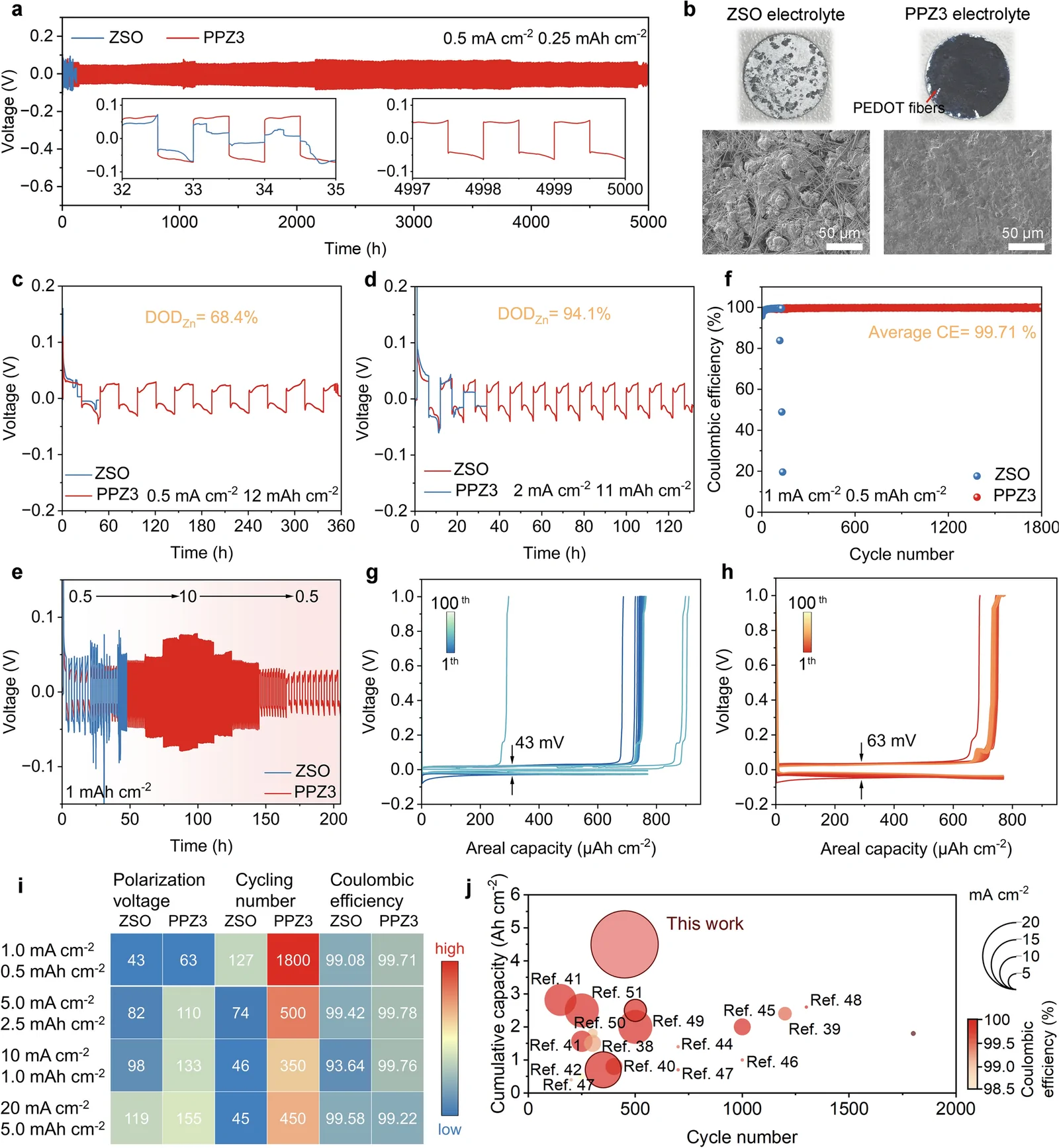
Long-term cycling tests of Zn symmetric cells with ZSO and PPZ3 electrolytes at **a** 0.5 mA cm^−2^/0.25 mAh cm^−2^. **b** SEM images of Zn anodes with the ZSO and PPZ3 electrolytes after cycling. The cycling performance of Zn symmetric cell at **c** 0.5 mA cm^−2^/12 mAh cm^−2^ and **d** 2 mA cm^−2^/11 mAh cm^−2^. **e** Rate performance of Zn||Zn symmetric cells. **f** Coulombic efficiency of Zn||Cu cells with the corresponding voltage-areal capacity profile in **g** ZSO and **h** PPZ3 electrolytes. **i** The heatmap of polarization voltage, cycling number and Coulombic efficiency of Zn asymmetric cells in various testing conditions. **j** Electrochemical performance Comparison of the PPZ3 electrolytes with other electrolytes

The practical effect of PPZ3 on Zn anode stability was evaluated in Zn||Zn symmetric cells. With PPZ3, the cell achieves a cycling lifespan of 5000 h at 0.5 mA cm^−2^ and 0.25 mAh cm^−2^ (Fig. 5a), nearly 156 times longer than with ZSO (32 h). SEM images of cycled Zn anodes show that PPZ3 leads to a uniform and dense surface (Fig. 5b), indicating stable deposition and dissolution, whereas ZSO leads to rampant dendrites. At a higher cycling capacity of 12 mAh cm^−2^ and a depth of discharge (DOD) of 68.4%, PPZ3 again enables longer and more stable cycling over 360 h than ZSO (Fig. 5c). Even at a high DOD of 94.1%, PPZ3 maintains better anode stability (Fig. 5d), highlighting its roles in improving Zn metal utilization and durability during repeated cycling. PPZ3 also supports stable cycling under harsher conditions of 10 mA cm^−2^ (Fig. S27). Moreover, Zn symmetric cells with PPZ3 exhibit high reversibility and stable operation under current densities varying from 0.5 to 10 mA cm^−2^ (Figs. 5e and S28). The superior cycling performance under diverse conditions confirms the efficacy of PPZ3 in optimizing the electrochemical performance of Zn anodes. Note that the DPPZ electrolyte is inadequate to improve the stability of Zn anodes (Fig. S29).

Similarly, in the Zn||Cu asymmetric cell, PPZ3 electrolyte enables highly stable and reversible Zn deposition/dissolution over 1800 cycles, with an average Coulombic efficiency of 99.71% at 1 mA cm^−2^ and 0.5 mAh cm^−2^, whereas cells with ZSO fail after only a few cycles (Fig. 5f). Voltage-areal capacity profiles further highlight this contrast that PPZ3 maintains a relatively higher and more stable voltage polarization throughout cycling compared to ZSO (Fig. 5g, h, respectively). CV curves of Zn||Cu asymmetric cells show that PPZ3 produces a larger nucleation overpotential and higher current densities for cathodic and anodic peaks than ZSO (Fig. S30). This observation indicates the PPZ3 electrolyte facilitates smaller, more uniform Zn nucleation and enhanced reaction kinetics for Zn deposition/dissolution. To further inspect the validity and superiority of the PPZ3 in enhancing reversibility of Zn deposition/dissolution, Zn||Cu cells were tested under more demanding conditions with current densities and capacities of 5 mA cm^−2^/2.5 mAh cm^−2^, 10 mA cm^−2^/1 mAh cm^−2^ and 20 mA cm^−2^/5 mAh cm^−2^ (Figs. S31-S33, respectively). A performance heatmap integrating polarization voltage, cycle number and Coulombic efficiency under these conditions clearly demonstrates the consistent reversibility and stability afforded by PPZ3 (Fig. 5i). This robustness is attributed to the modified Zn^2+^ solvation structure, facilitated ion transport and effective suppression of side reactions. In contrast, ZSO leads to short cycle life and fluctuating Coulombic efficiency, reflecting the intrinsic instability of Zn in weakly acidic salt solutions. The cycling lifespan, cumulative plating capacity and Coulombic efficiency achieved with PPZ3 also exceed those of most previously reported electrolyte systems [38–51] (Fig. 5j). These results confirm that the PPZ3 biphasic electrolyte significantly improves the reversibility of Zn deposition/dissolution and stabilizes the electrode–electrolyte interface.

### Electrochemical Performance of Full Cells

**Fig. 6:**
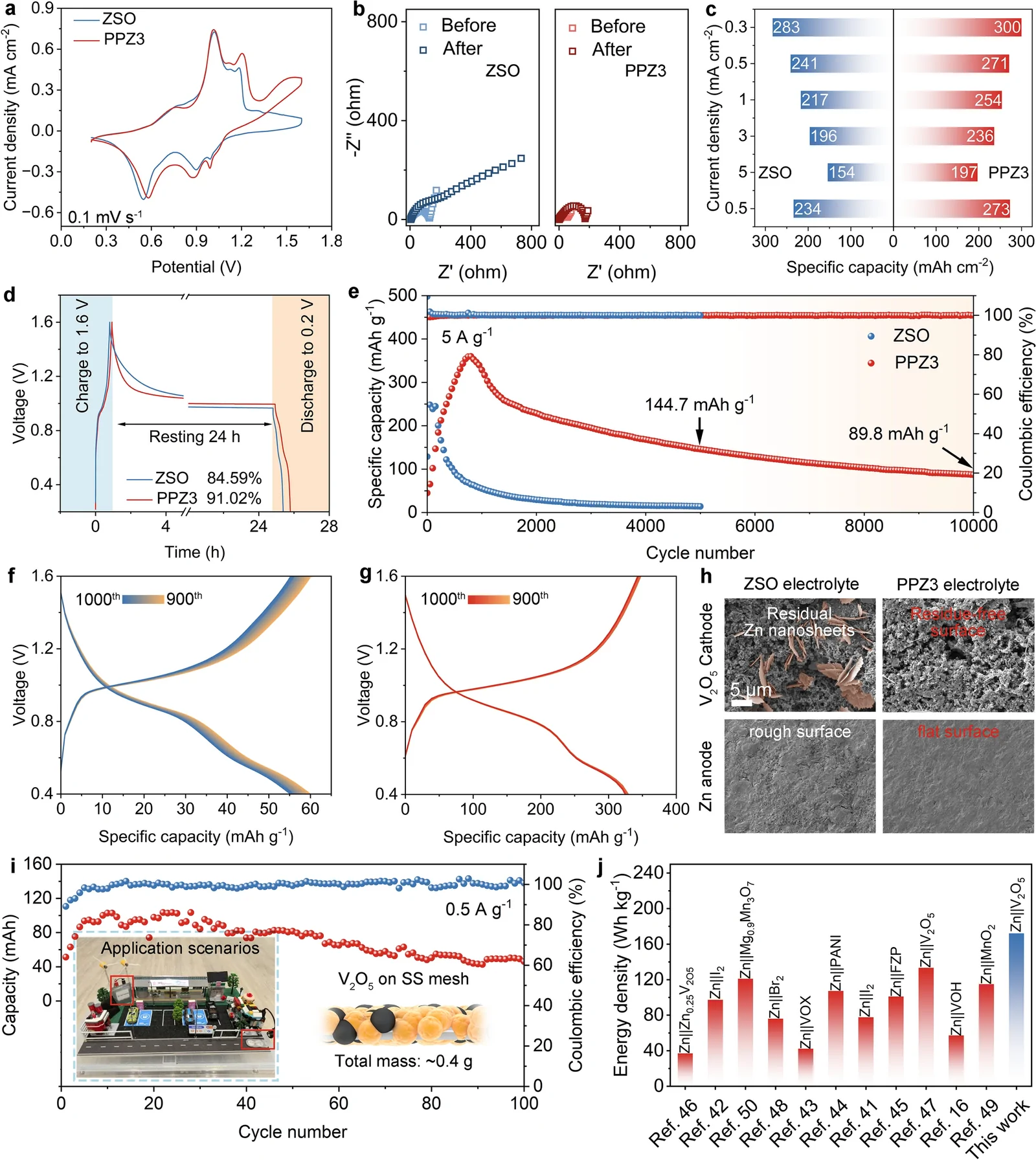
Electrochemical performance of Zn||V_2_O_5_ cells with various electrolytes. **a** CV curves at scan rate of 0.1 mV s^−1^. **b** EIS plots of Zn||V_2_O_5_ cells before and after cycling at 0.1 A g^−1^. **c** Electrochemical test of Rate performance at various current densities. **d** Capacity retention test after resting 24 h. **e** Cycling performance of cells at 5 A g^−1^ with the voltage profile using the **f** ZSO and **g** PPZ3 electrolyte at the selected cycles. **h** SEM images of electrodes using the ZSO and PPZ3 electrolytes after cycling. **i** The cycling performance of Zn||V_2_O_5_ pouch cell with the PPZ3 electrolyte. **j** Performance comparison of the pouch cells

Vanadium pentoxide (V_2_O_5_) as a prime cathodic material candidate of AZIBs was used to assemble full cells for evaluating the practical feasibility of PPZ3 electrolyte (Fig. S34). The V_2_O_5_ material synthesized by heat sintering was characterized by XRD (Fig. S35) and SEM (Fig. S36). CV curves of Zn||V_2_O_5_ full cells show similar redox peaks in both ZSO and PPZ3 (Fig. 6a), but the current densities for anodic and cathodic reactions are notably higher in PPZ3, indicating enhanced electrochemical reactivity for Zn^2+^ insertion/extraction. Additionally, the cell containing PPZ3 electrolyte shows reduced internal resistance both before and after 50 cycles (Fig. 6b). Benefiting from the fast ion transport, high reaction kinetics and low internal resistance enabled by PPZ3, Zn||V_2_O_5_ full cell delivers high specific capacities of 300, 271, 254, 236, and 197 mAh g^−1^ at current densities ranging from 0.3 to 5 A g^−1^ (Figs. 6c and S37), outperforming the ZSO system. The PPZ3-based cell also exhibits a superior self-discharge capacity retention of 91.02% (Fig. 6d), compared to 84.59% of ZSO, underscoring its effectiveness in suppressing parasitic reactions. Furthermore, the PPZ3 cell outputs higher discharge capacity at the current density of 0.5 A g^−1^, compared to ZSO electrolyte (Fig. S38). At higher current density of 5 A g^−1^, the PPZ3 still demonstrates outstanding cycling performance, retaining 144.7 mAh g^−1^ after 5000 cycles and 89.8 mAh g^−1^ after 10,000 cycles at 5 A g^−1^ (Fig. 6e). However, the ZSO cell exhibits rapid capacity decay (Fig. 6f, g). SEM images disclose that after cycling, the ZSO generates severe "dead Zn" and Zn nanosheet accumulation on both V_2_O_5_ cathode and Zn electrodes (Fig. 6h), a consequence of poor deposition/dissolution reversibility. In comparison, electrode surfaces remain smooth and clean in the PPZ3 system. The practical utility of PPZ3 was further validated in Zn||V_2_O_5_ pouch cells and the mass of cell components is shown in Table S2. A three-cell series configuration delivered an open-circuit voltage of 4.28 V and reliably powered a fan (Fig. S39). The PPZ3 pouch cell also provided an average discharge capacity of 72.5 mAh with an energy density of 172.2 Wh kg^−1^ based on the mass of active material (Fig. 6i), outrunning previously reported advanced AZIB prototypes [52–62] (Fig. 6j). The excellent cycling performance of Zn||V_2_O_5_ cells confirms the practical effectiveness and feasibility of the designed PPZ3 electrolyte for large-scale AZIB applications.

## Conclusions

In summary, we develop a self-separating biphasic electrolyte through the chemical–mechanical dissociation of PP in a ZSO solution. In this system, the PSS polymer chains bearing negatively charged sulfonic groups remain dissolved in the electrolyte, whereas the insoluble PEDOT chains with delocalized conjugated π electrons spontaneously separate from the system. Owing to its distinctive structure, the biphasic electrolyte not only remodels the Zn^2+^ solvation structure but also forms an electron-rich interphase on the electrode, which facilitates ion transport and reduction and repels SO_4_^2−^ anions. These attributes collectively promote rapid ion desolvation, suppress side reactions and enable uniform Zn deposition along the Zn(101) crystal plane. As a result, Zn anodes employing this biphasic electrolyte exhibit stable operation with high average Coulombic efficiencies under various cycling conditions. Even at high DODs of 68.4% and 94.1%, this electrolyte sustains excellent cycling stability and longevity. Its full cells also demonstrate remarkable cyclability with high capacities of 144.7 mAh g^−1^ at 5000 cycles and 89.8 mAh g^−1^ at 10,000 cycles.

## Supplementary Information

Below is the link to the electronic supplementary material.

Supplementary file1 (DOCX 8525 kb)
