# Supplementary material for: Self-Separating Biphasic Electrolyte Enables High-Performance Aqueous Zinc-Ion Batteries via Electron-Enriched Interphase Engineering
Source: Nanomicro Lett. 2026 May 12;18:367. doi: 10.1007/s40820-026-02219-3 (PMC13168403; doi:10.1007/s40820-026-02219-3)
Supplement: Supplementary file 1 — Supplementary file1 (DOCX 8525 kb) [file 40820_2026_2219_MOESM1_ESM.docx]

Supporting Information for

**Self-Separating** **Biphasic Electrolyte Enables High-Performance Aqueous Zinc-Ion Batteries via Electron-Enriched Interphase Engineering**

Chengwu Yang^1^, Pattaraporn Woottapanit^2,3^, Qizhi Hou^1^, Zhiqiang Dai^1^, Wanwisa Limphirat^4^, Jiaqian Qin^2,5,6*^, Xinyu Zhang^1,2*^

^1^State Key Laboratory of Metastable Materials Science and Technology, Yanshan University, Qinhuangdao 066004, P. R. China

^2^Department of Materials Science, Faculty of Science, Chulalongkorn University, Bangkok 10330, Thailand

^3^International Graduate Program of Nanoscience & Technology (Interdisciplinary), Graduate School, Chulalongkorn University, Bangkok, 10330, Thailand

^4^Synchrotron Light Research Institute (Public Organization), Nakhon Ratchasima 30000, Thailand

^5^Center of Excellence in Responsive Wearable Materials, Chulalongkorn University, Bangkok 10330, Thailand

^6^Energy Research Institute, Chulalongkorn University, Bangkok, 10330, Thailand

*Corresponding authors. E-mail: [jiaqian.q@chula.ac.th](mailto:jiaqian.q@chula.ac.th) (Jiaqian Qin); [xyzhang@ysu.edu.cn](mailto:xyzhang@ysu.edu.cn) (Xinyu Zhang)

**Supplementary Figures and Tables**


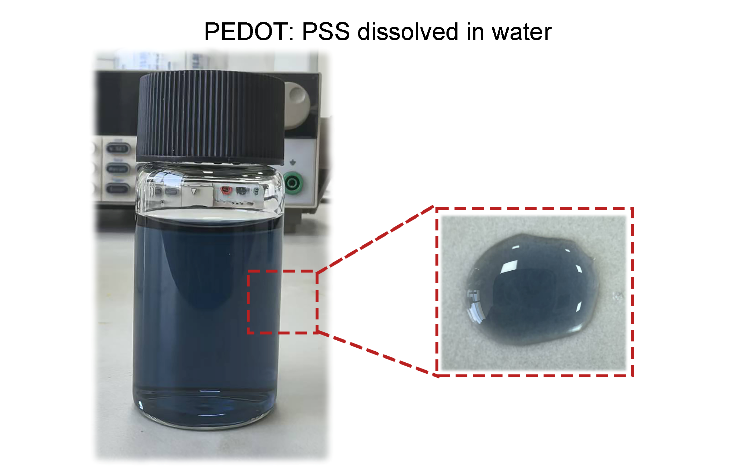


**Fig. S1** The digital photos of PP in water.


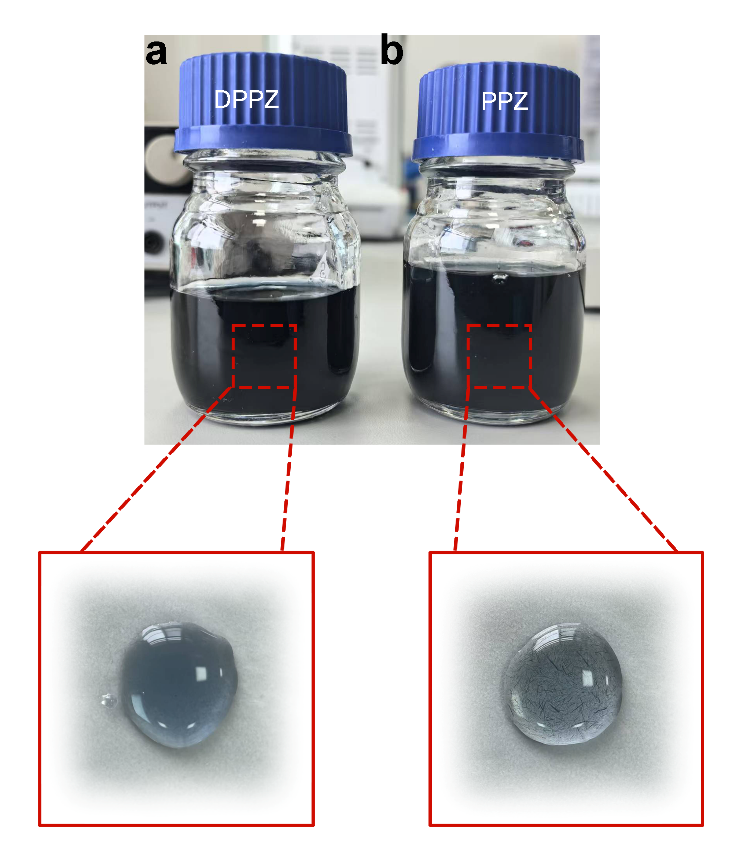


**Fig. S2** The digital photos of **a** DPPZ and **b** PPZ solutions.


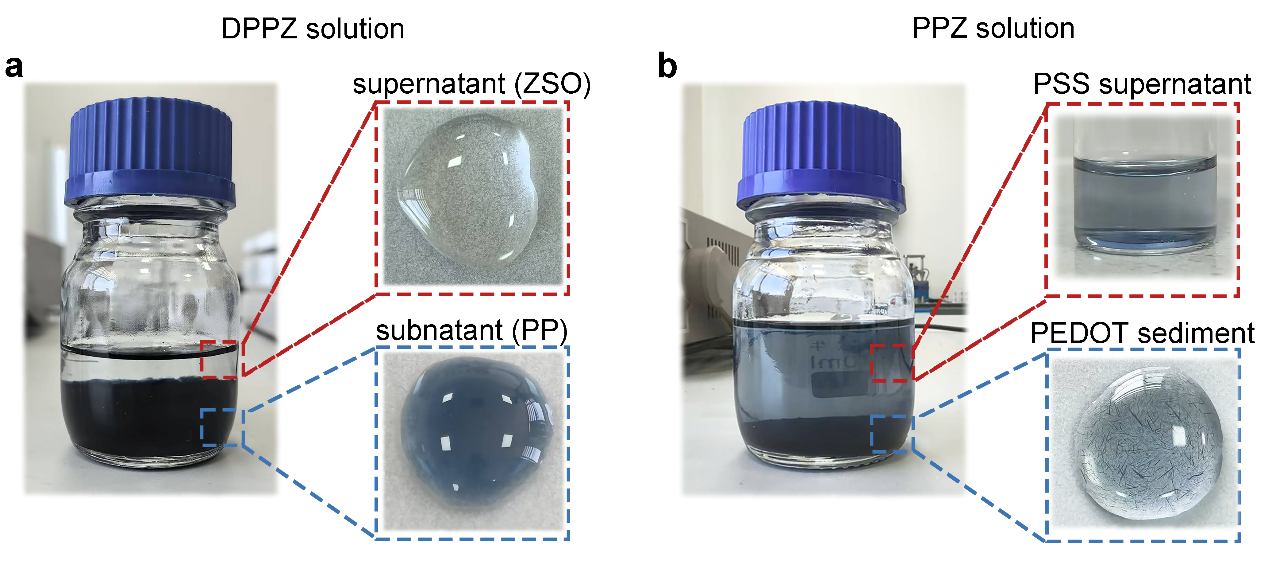


**Fig. S3** The digital photos of **a** DPPZ and **b** PPZ solutions after 24 h sedimentation with the corresponding supernatants and subnatants.


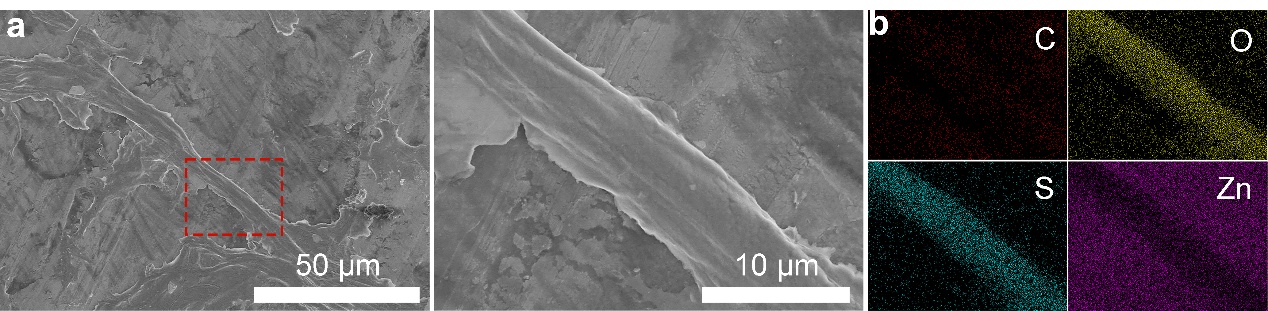


**Fig. S4** **a** SEM images of PEDOT fiber on Zn substrate with **b** the corresponding EDS mapping of C, O S and Zn elements.


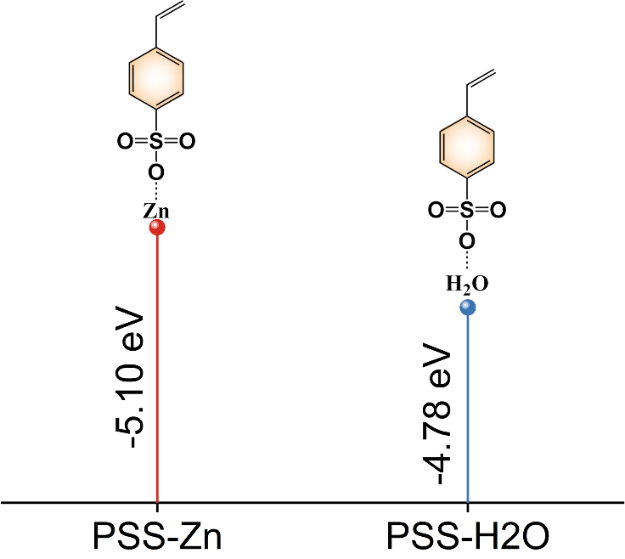


**Fig. S5** The binding energy of PSS with Zn^2+^ ion and water molecule.


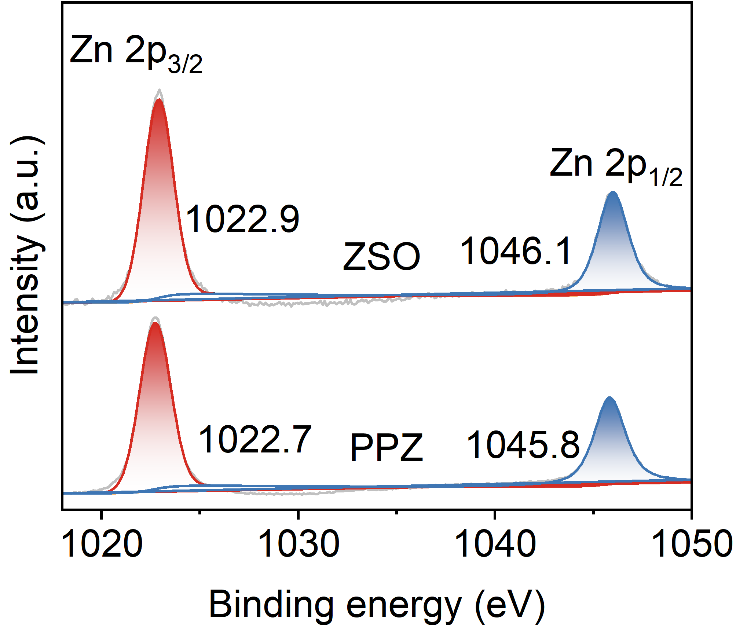


**Fig. S6** XPS spectra of the ZSO and PPZ.


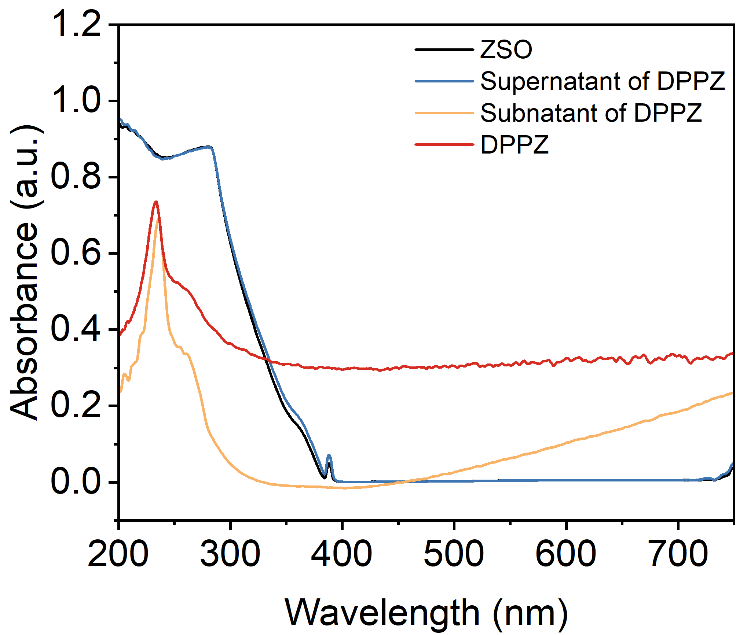


**Fig. S7** UV-VIS absorption spectra of the samples. The UV-VIS absorption of supernatant of DPPZ is similar with that of ZSO. Subnatant of DPPZ also shows similar light absorption with DPPZ solution. These results indicates that PEDOT and PSS are not separated in the DPPZ solution.


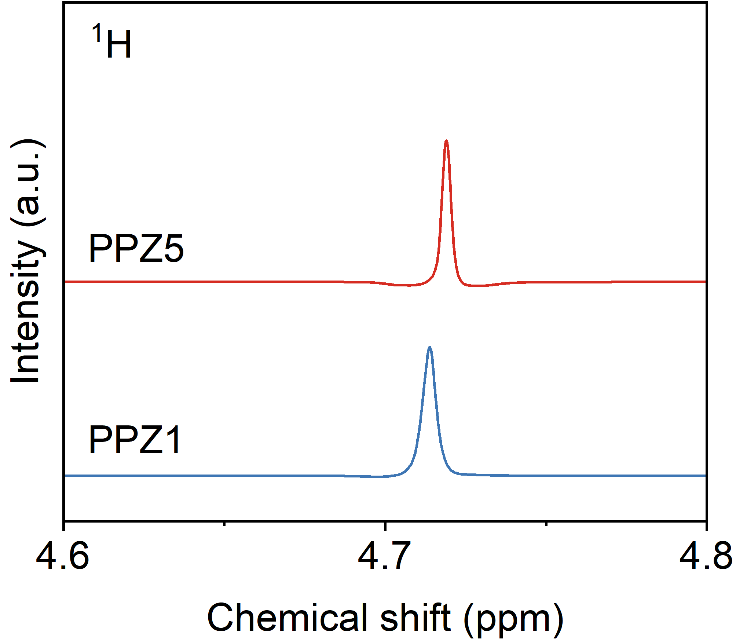


**Fig. S8** 1H NMR spectra of PPZ1 and PPZ5 electrolytes.


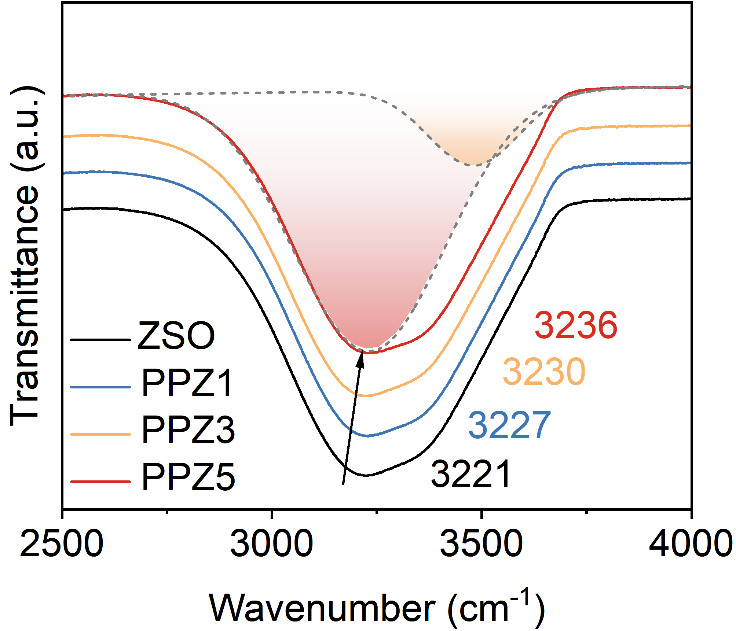


**Fig. S9** FTIR spectra of the ZSO and biphasic electrolytes.


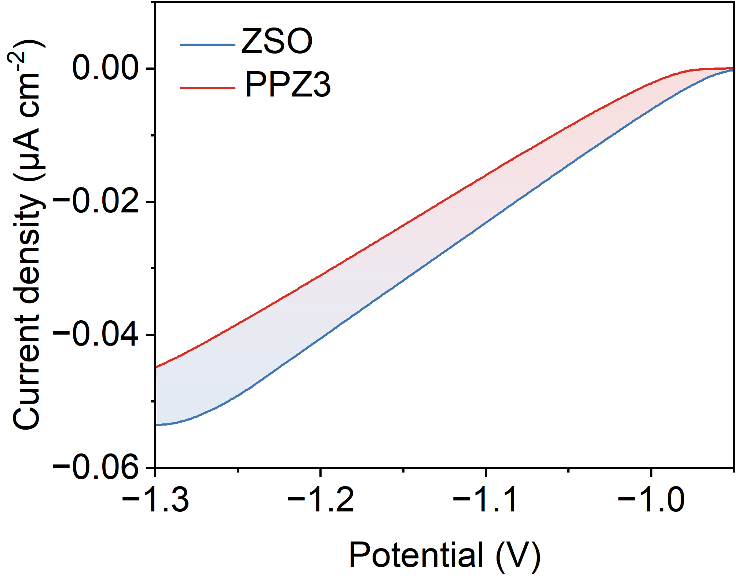


**Fig. S10** LSV curves of the ZSO and PPZ3 electrolytes.


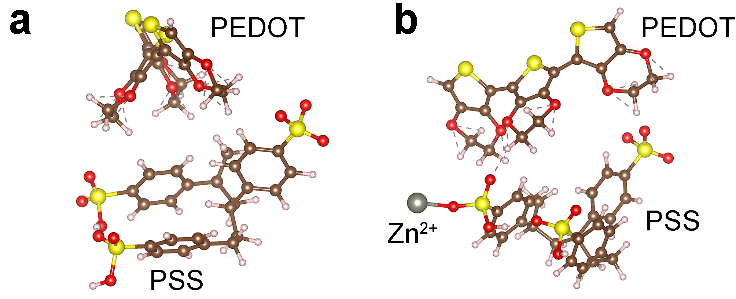


**Fig. S11** The structural models for ESP calculations. **a** PEDOT + PSS complex and **b** PEDOT + PSS complex combined with one Zn^2+^ ion.


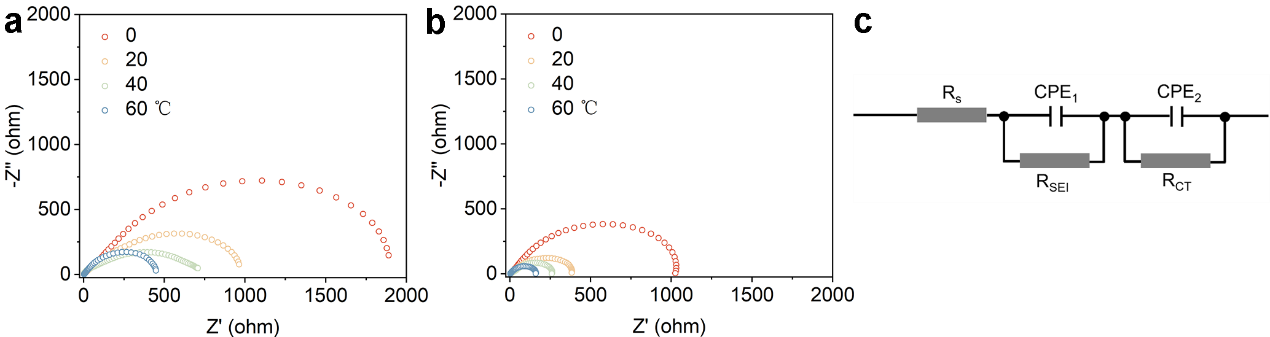


**Fig. S12** EIS plots of Zn||Zn symmetric cells with **a** the ZSO and **b** PPZ3 at various temperatures for the calculation of ionic desolvation energy. **c** The equivalent circuit of EIS plots.


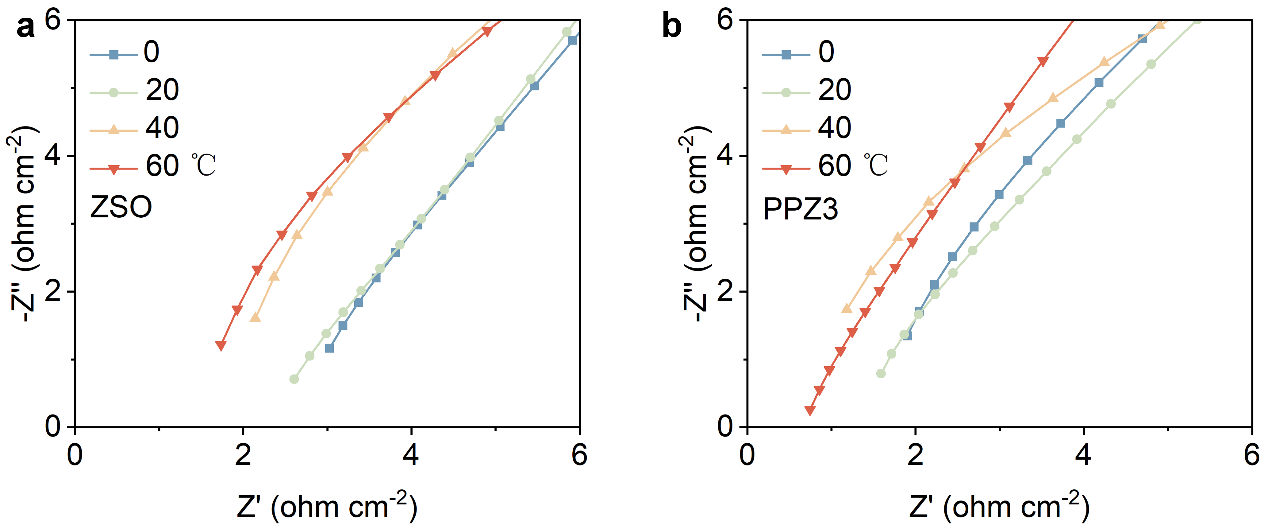


**Fig. S13** EIS plots of Zn||Zn symmetric cells with **a** the ZSO and **b** PPZ3 at various temperatures for the calculation of ionic conductivity.


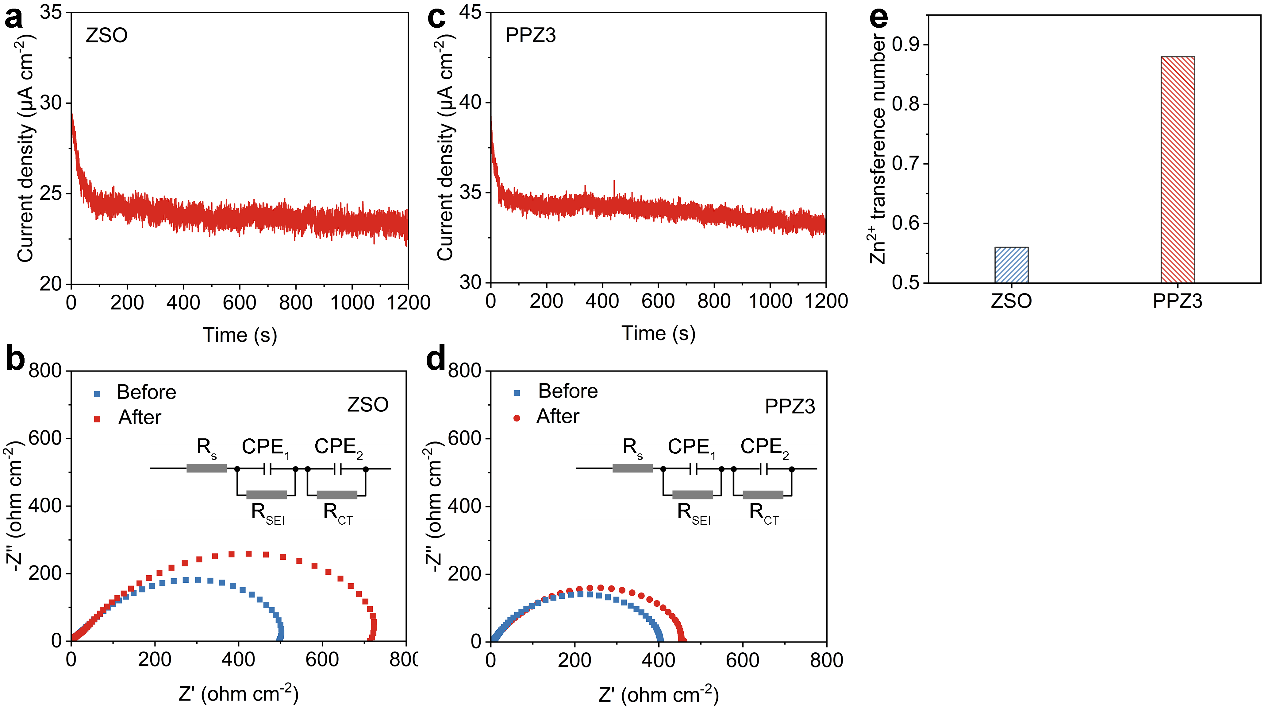


**Fig. S14** I-t curves and the corresponding EIS plots of the Zn symmetric cells with **a, b** the ZSO and **c, d** PPZ3 electrolytes. **e** The calculated Zn^2+^ transference number.


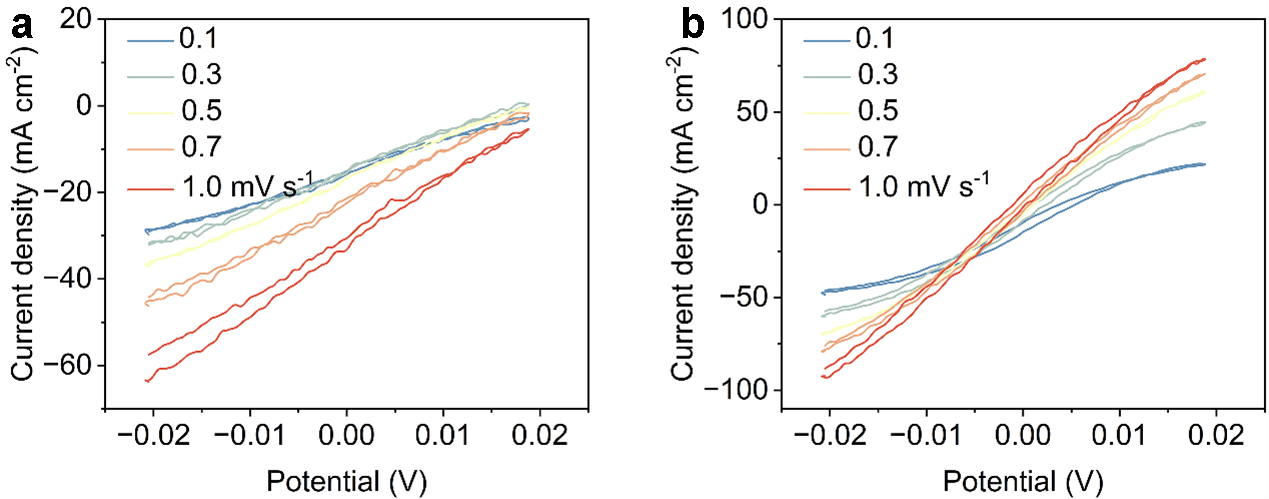


**Fig. S15** CV curves of Zn||Zn symmetric cells with **a** the ZSO and **b** PPZ3 electrolytes at the scan rate range of 0.1 to 1 mV s^-1^.


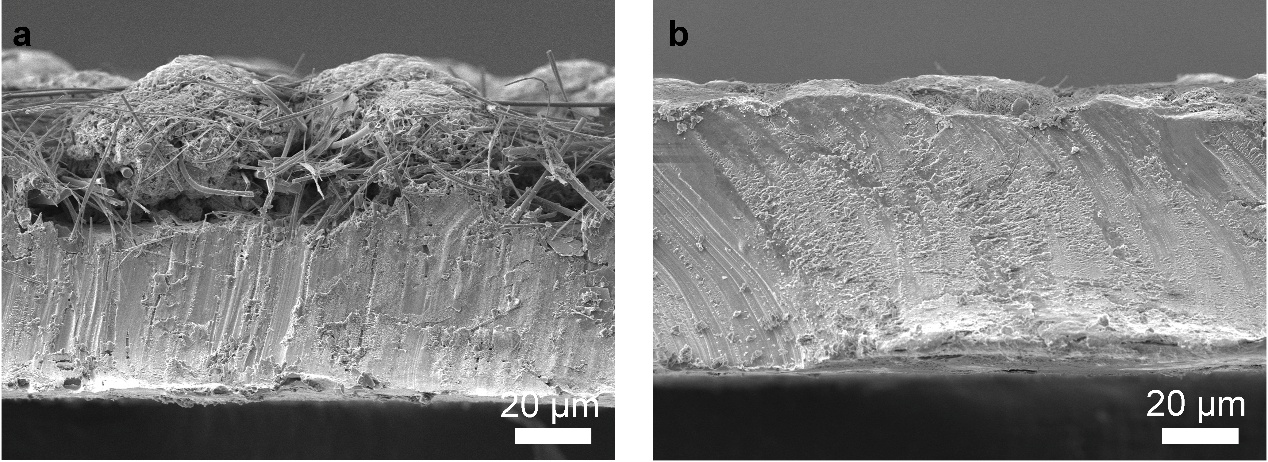


**Fig. S16** SEM images of Cross-sectional view of Zn anodes after Zn deposition at the **a** ZSO and **b** PPZ3 electrolytes.


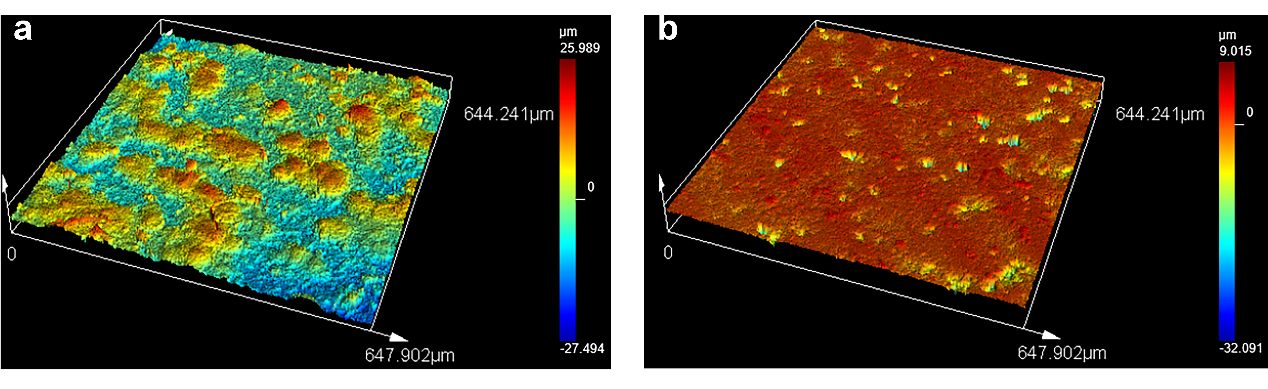


**Fig. S17** The confocal images of Zn anode after deposition in the electrolytes of **a** ZSO and **b** PPZ3.

*
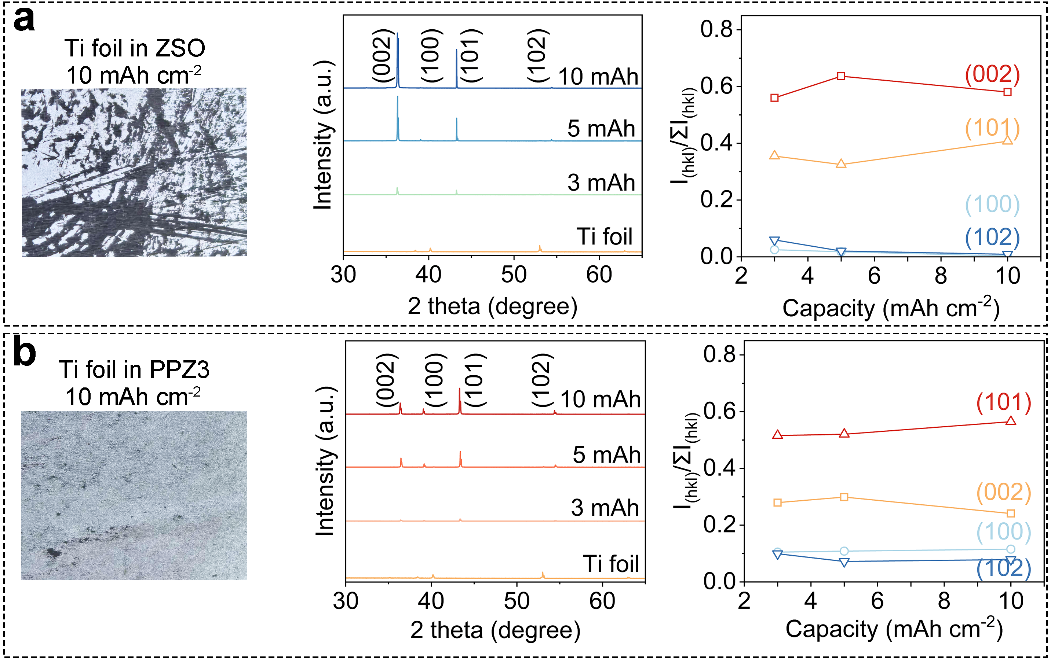
*

**Fig. S18** XRD patterns and relative texture coefficients of Zn deposited on Ti foil with the corresponding digital photo. **a** ZSO and **b** PPZ3 electrolytes.


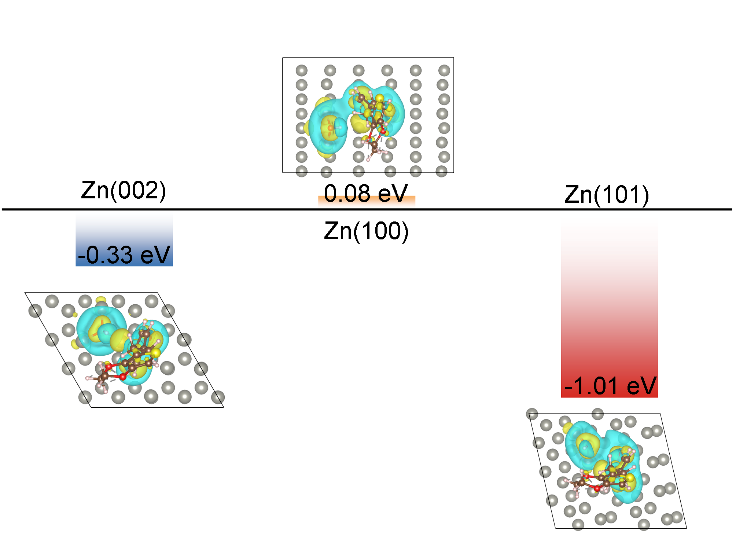


**Fig. S19** The adsorption energy of PEDOT + PSS on Zn(002), Zn(100) and Zn(101).


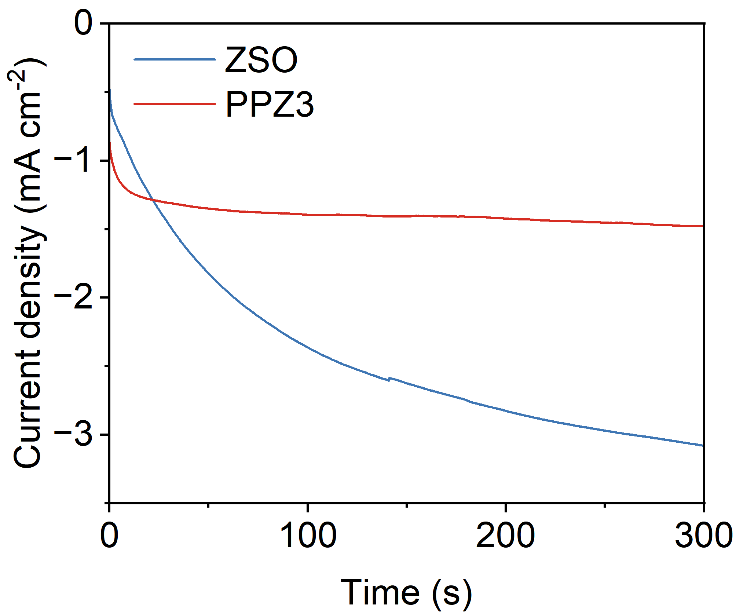


**Fig. S20** I-t curves of the Zn symmetric cells with different electrolyte tested at -150 mV.

*
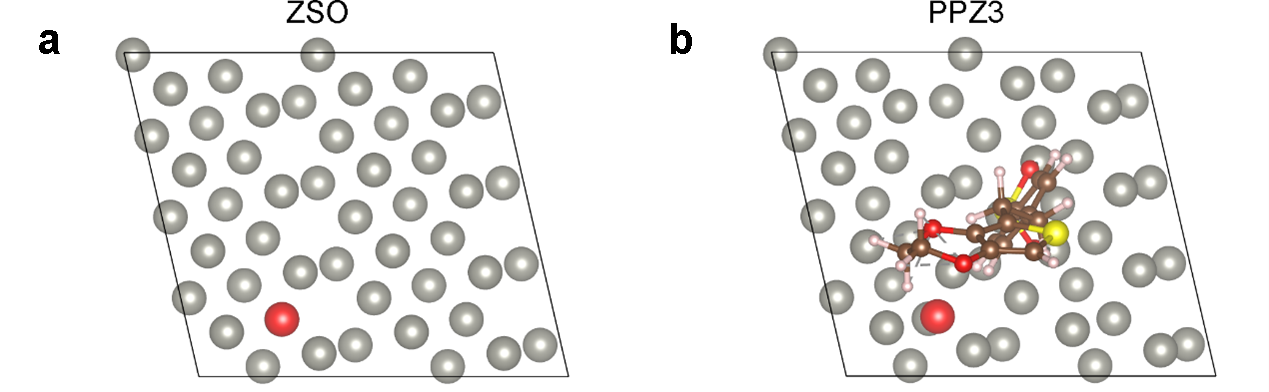
*

**Fig. S21** The initial configuration of Zn^2+^ migration on **a** ZSO and **b** PPZ3 electrolytes.


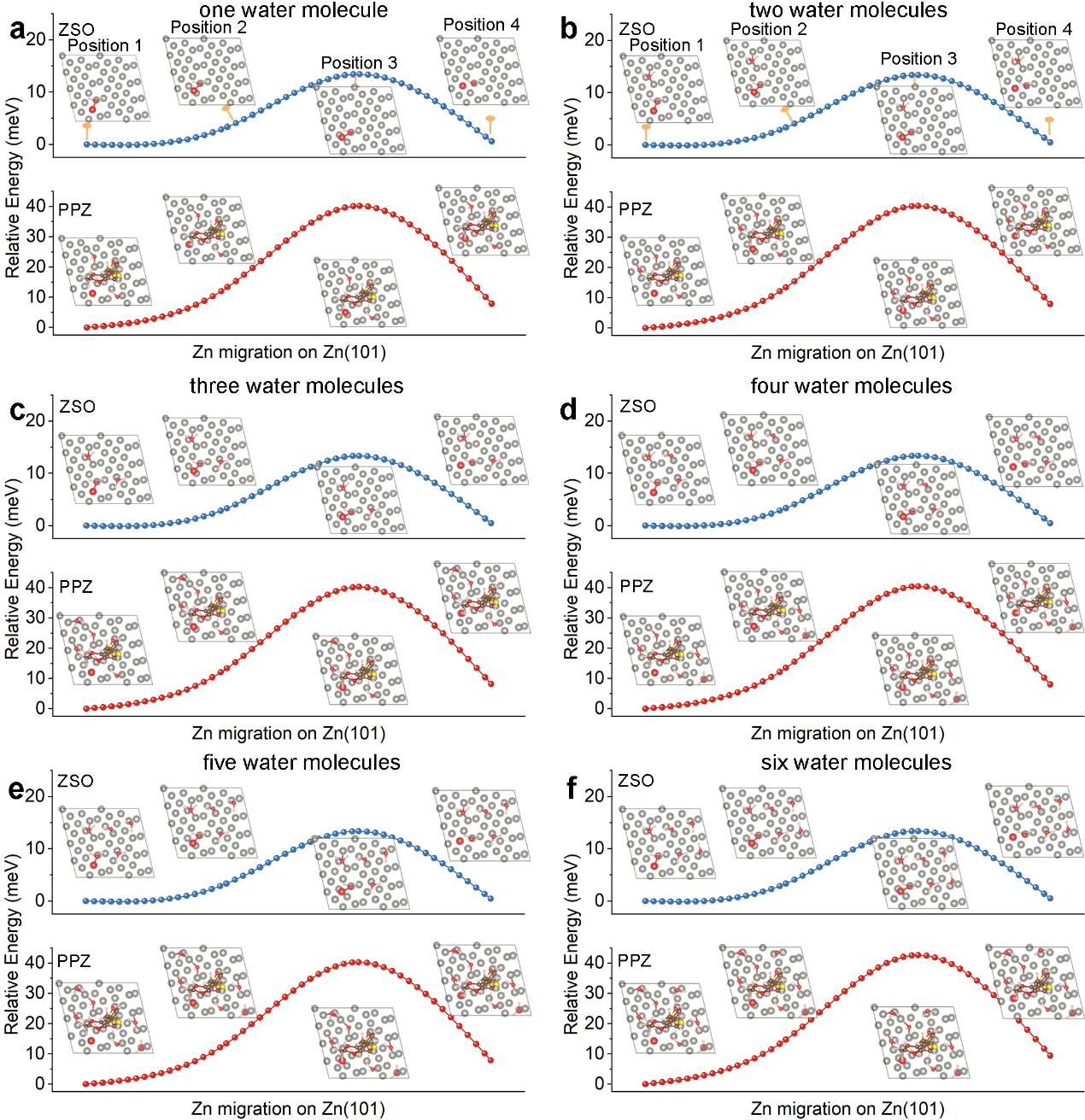


**Fig. S22** The simulations of Zn^2+^ migration on the Zn surface with **a** one, **b** two, **c** three, **d** four, **e** five and **f** six water adsorptions.


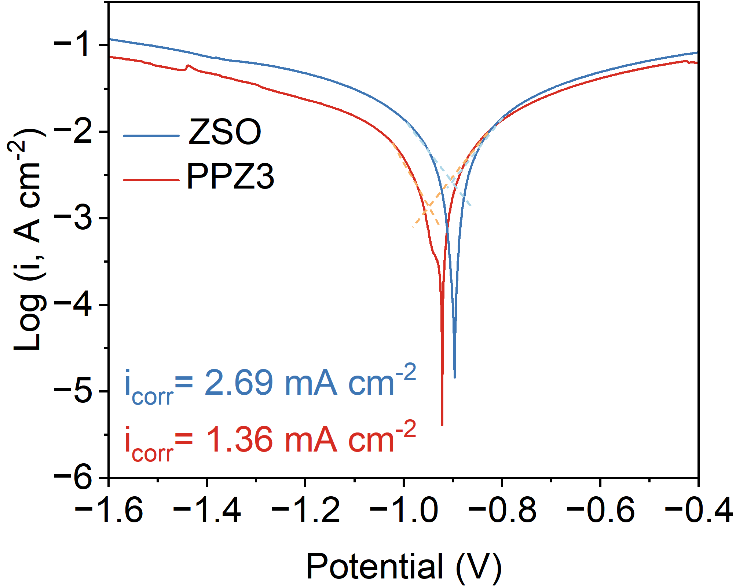


**Fig. S23** Tafel curves of the ZSO and PPZ3 electrolytes.


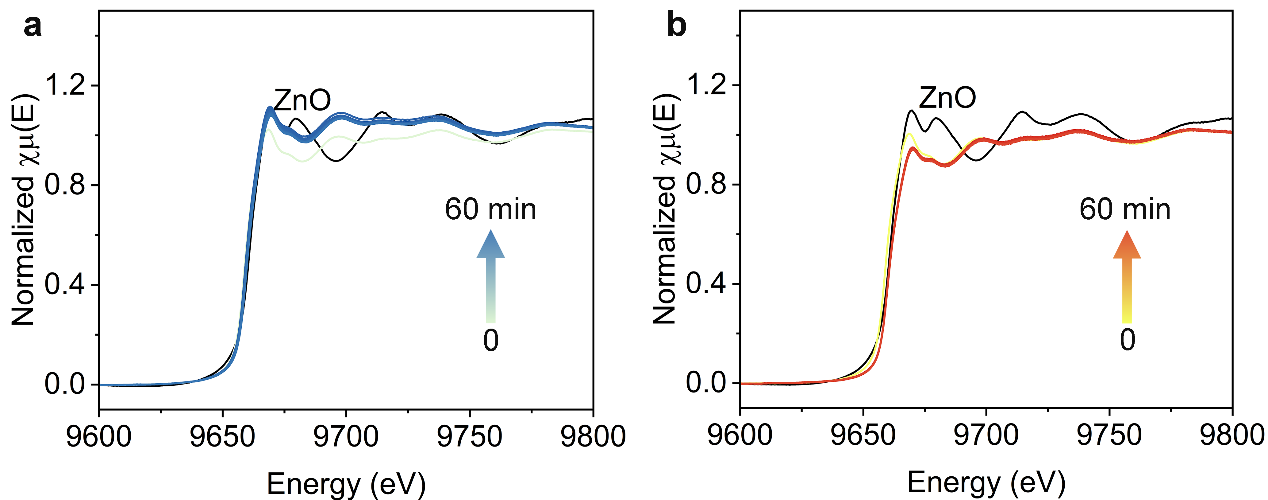


**Fig. S24** In-situ XAS spectra of Zn anode with **a** ZSO and **b** PPZ3 electrolytes during Zn deposition.


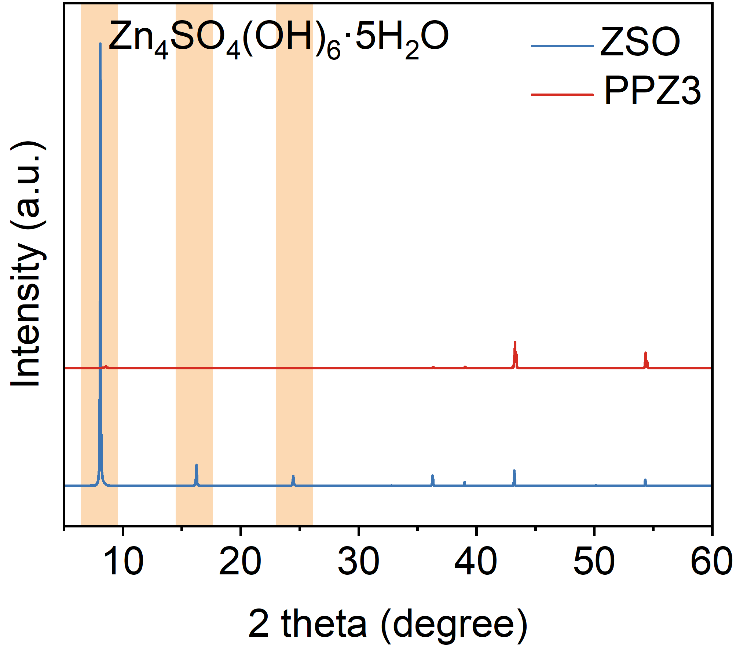


**Fig. S25** XRD patterns of Zn anodes after cycling.


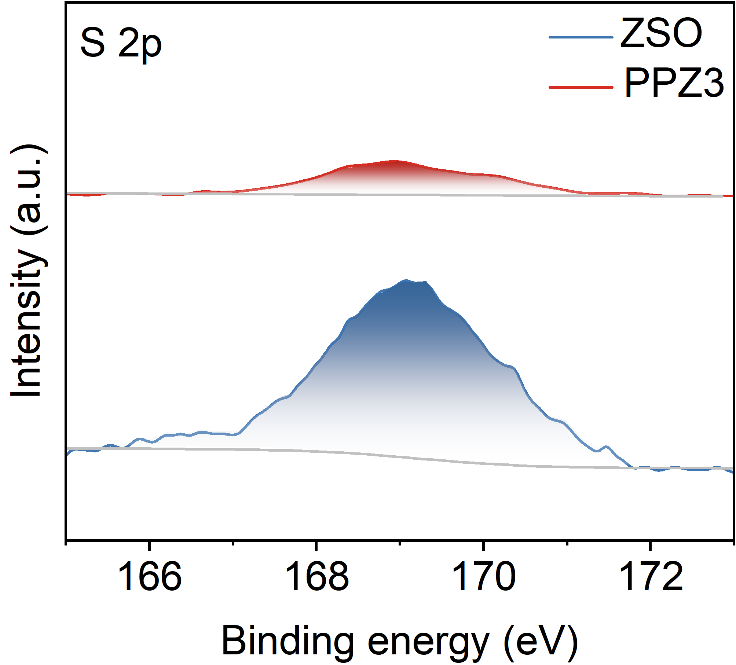


**Fig. S26** The S 2p XPS spectra of Zn anodes after cycling in the different electrolytes.


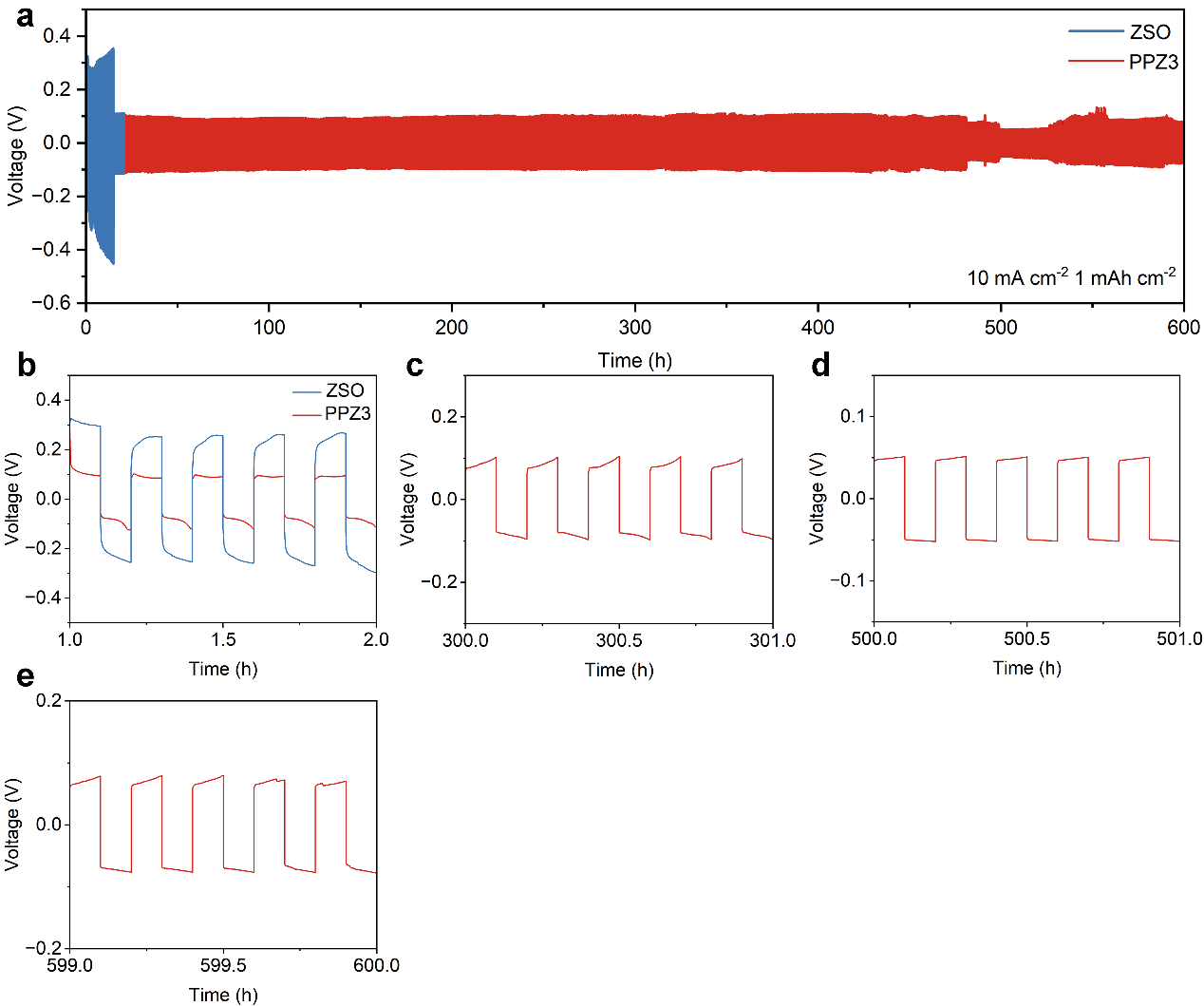


**Fig. S27** **a** The cycling performance of Zn||Zn symmetric cells with ZSO and PPZ3 electrolytes at 10 mA cm^-2^/1 mAh cm^-2^. The voltage-time profile of the symmetric cells at **b** 1-2, **c** 300-301, **d** 500-501 and **e** 599-600 h.


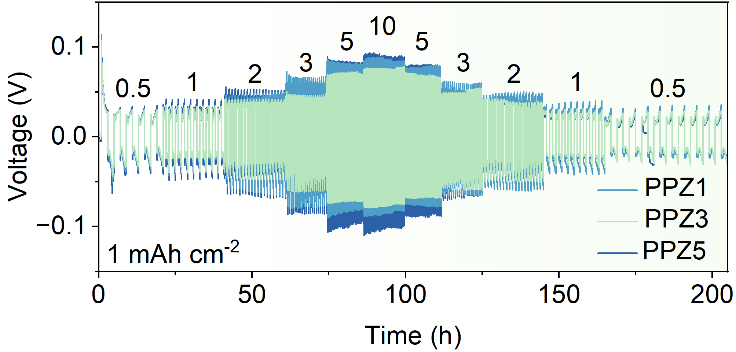


**Fig. S28** Rate performance of Zn||Zn symmetric cells with the PPZ electrolytes.


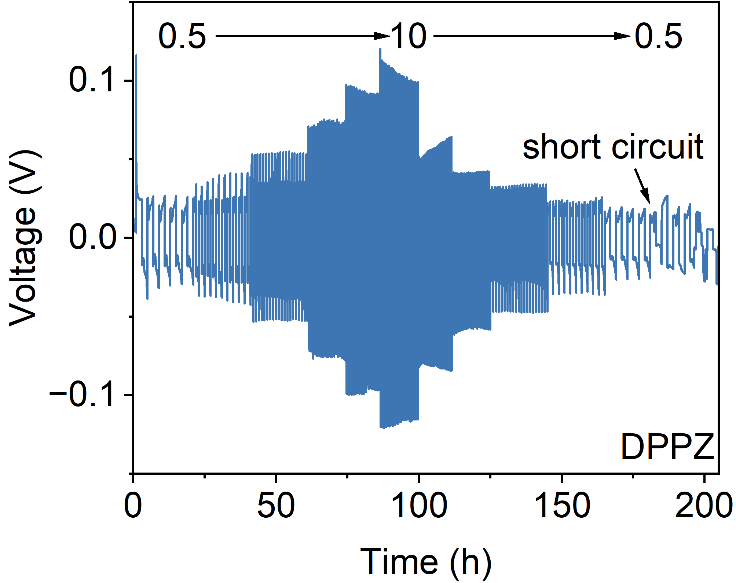


**Fig. S29** Rate performance of Zn||Zn symmetric cells with DPPZ electrolyte.


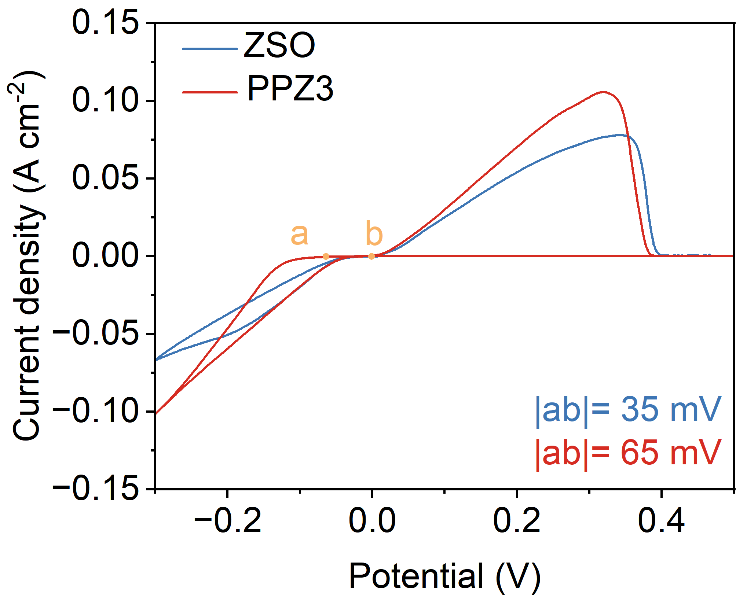


**Fig. S30** CV curves of Zn||Cu asymmetric cell with different electrolytes.


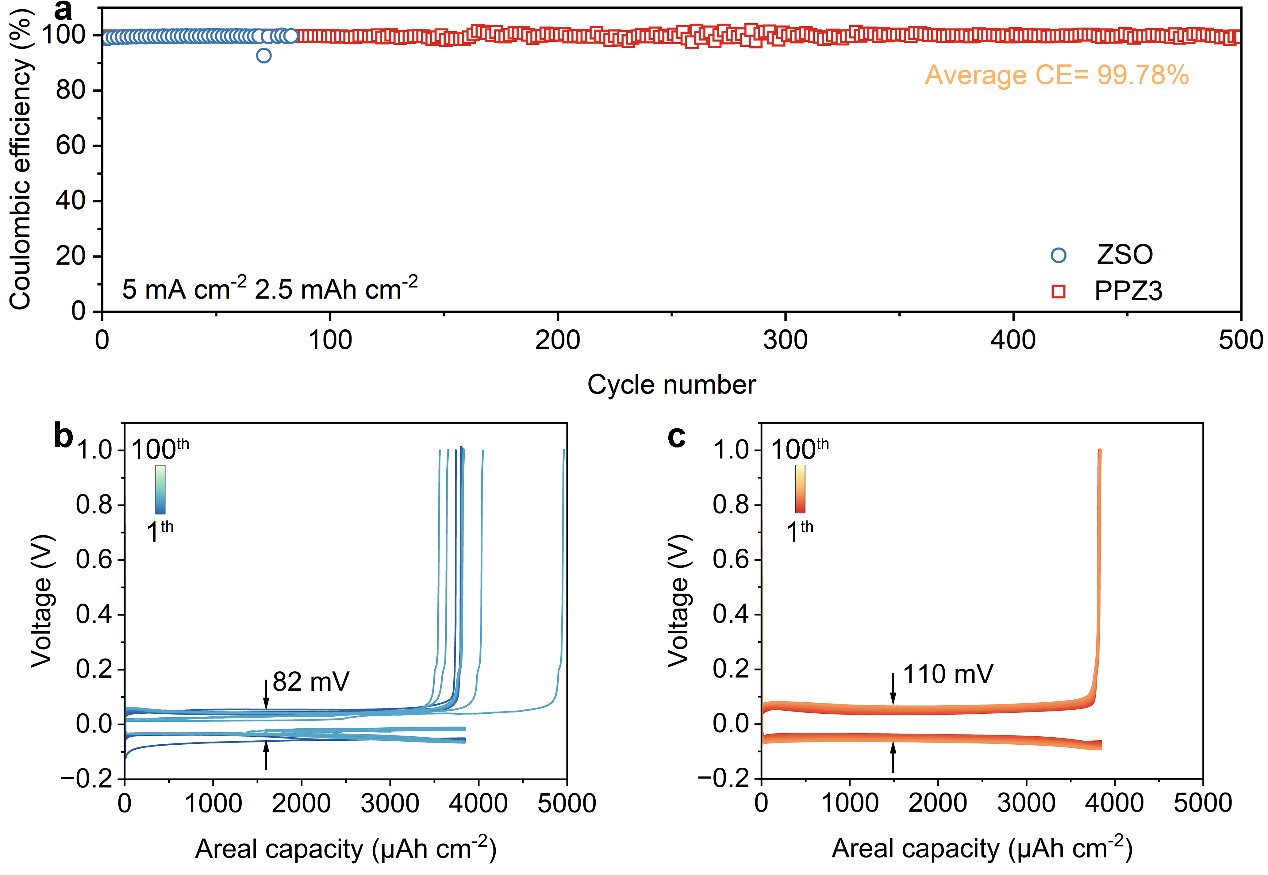


**Fig. S31** **a** The cycling performance of Zn||Cu asymmetric cells with ZSO and PPZ3 electrolytes at 5 mA cm^-2^/2.5 mAh cm^-2^. The voltage-areal capacity profile of the asymmetric cells with **b** ZSO and **c** PPZ3.


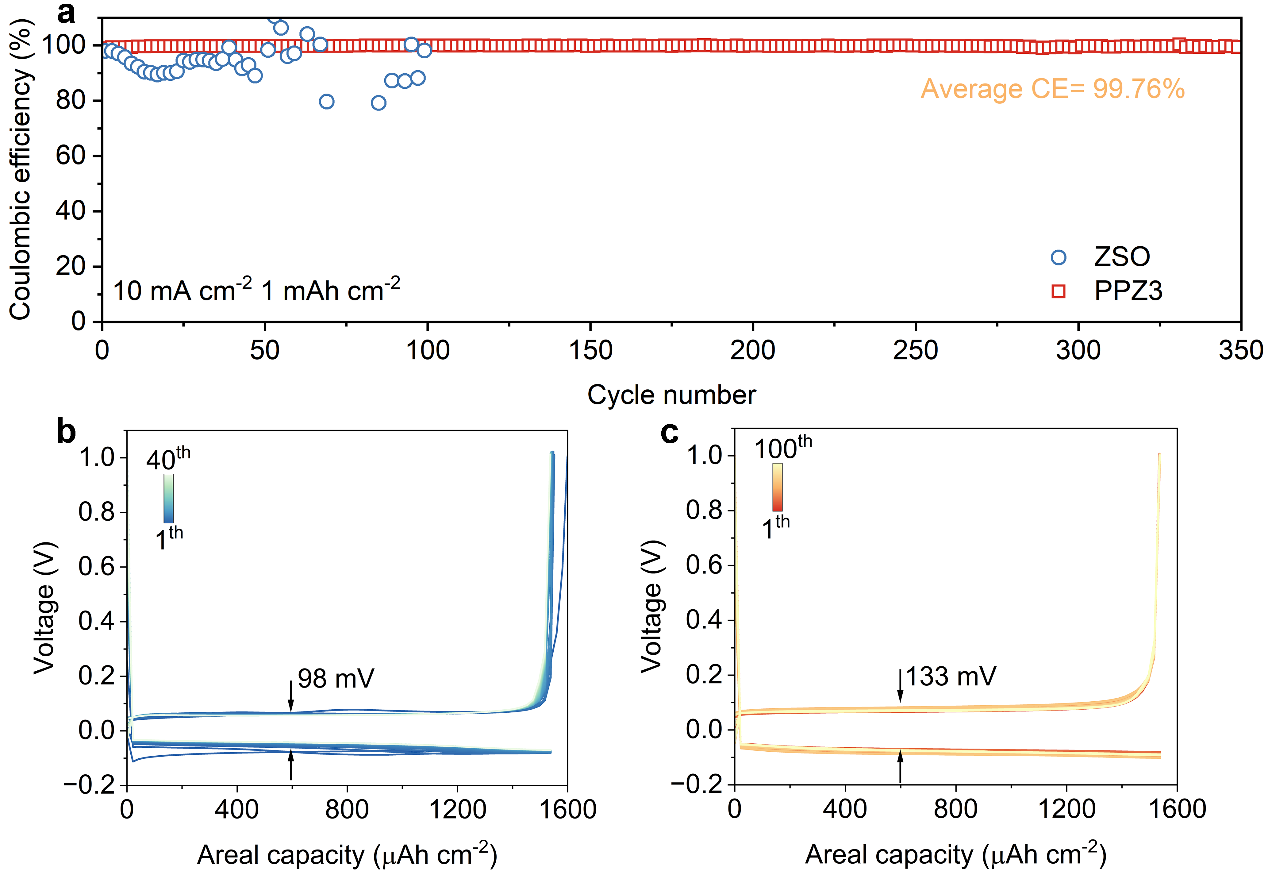


**Fig. S32** **a** The cycling performance of Zn||Cu asymmetric cells with ZSO and PPZ3 electrolytes at 10 mA cm^-2^/1 mAh cm^-2^. The voltage-areal capacity profile of the asymmetric cells with **b** ZSO and **c** PPZ3.


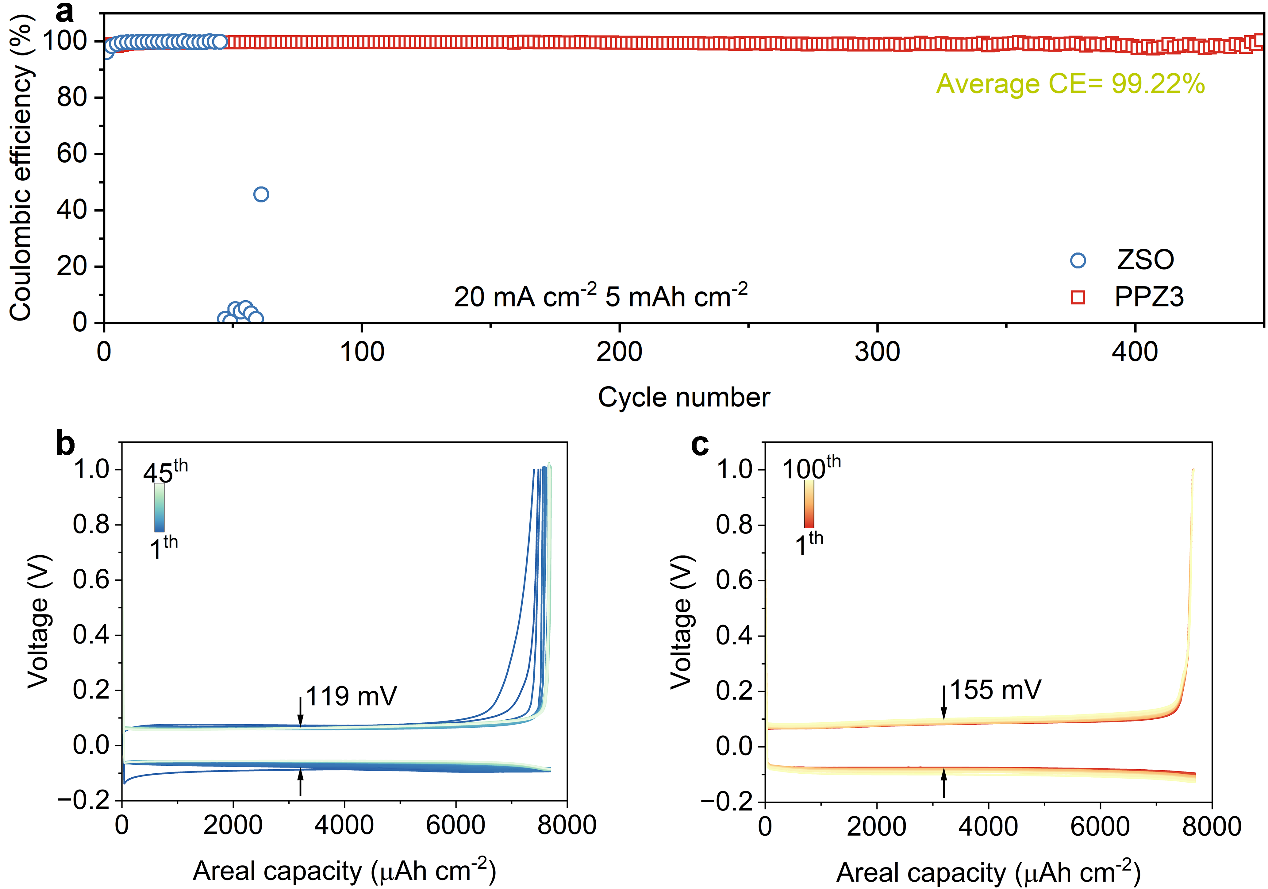


**Fig. S33** **a** The cycling performance of Zn||Cu asymmetric cells with ZSO and PPZ3 electrolytes at 20 mA cm^-2^/5 mAh cm^-2^. The voltage-areal capacity profile of the asymmetric cells with **b** ZSO and **c** PPZ3.


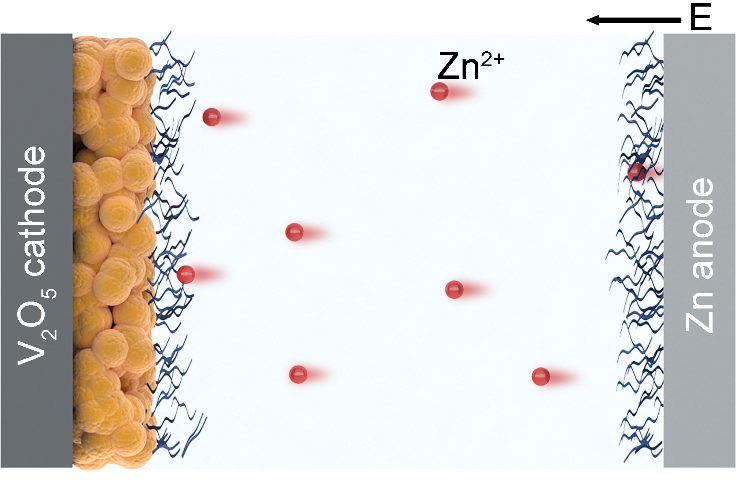


**Fig. S34** Schematical illustration of Zn||V_2_O_5_ full cell with the PPZ3 electrolyte.


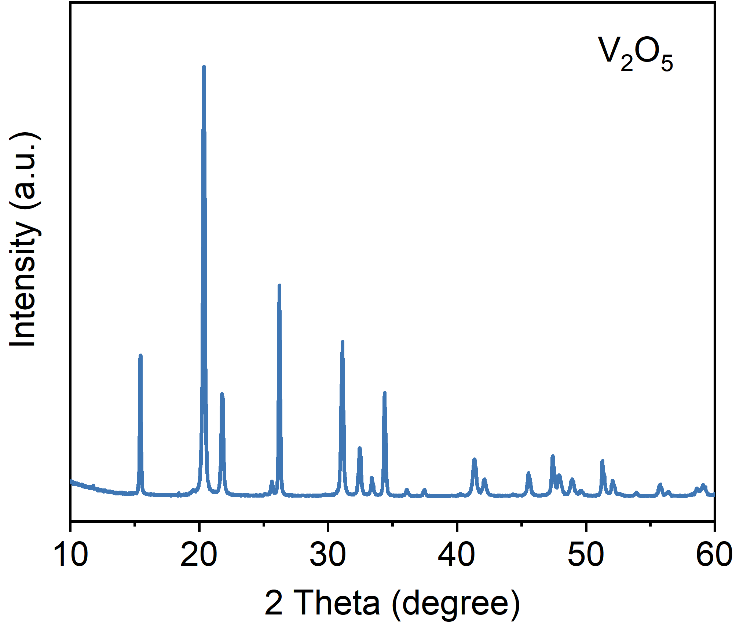


**Fig. S35** XRD pattern of V_2_O_5_ cathode material.


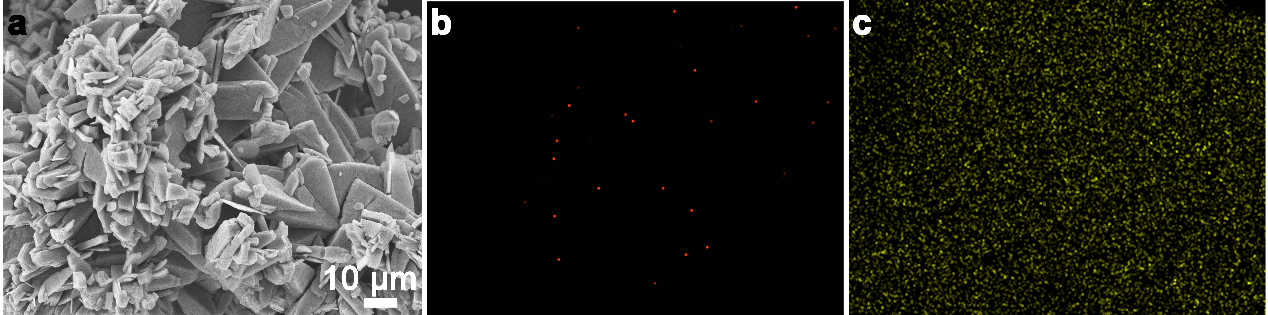


**Fig. S36** **a** SEM image of V_2_O_5_ cathode material with the corresponding elemental mapping of **b** O and **c** V elements.


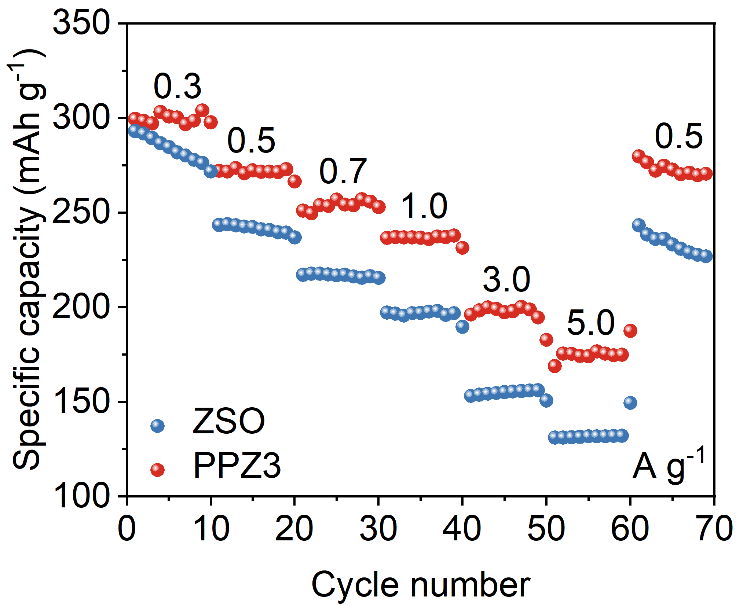


**Fig. S37** The rate performance of Zn||V_2_O_5_ full cells with ZSO and PPZ3 electrolytes at the current density range from 0.3-5 A g^-1^.


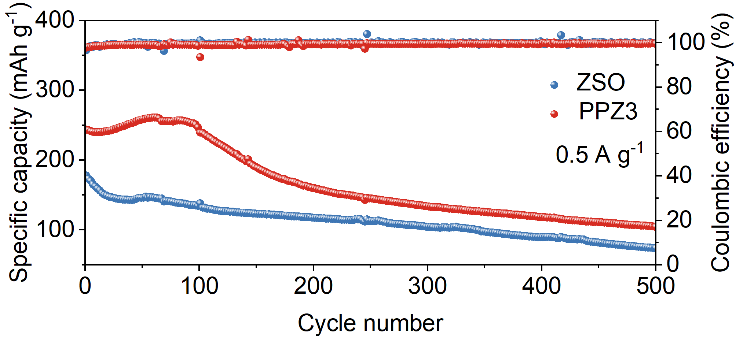


**Fig. S38** Long-term cycling tests of ZSO and PPZ3 full cells at 0.5 A g^-1^.


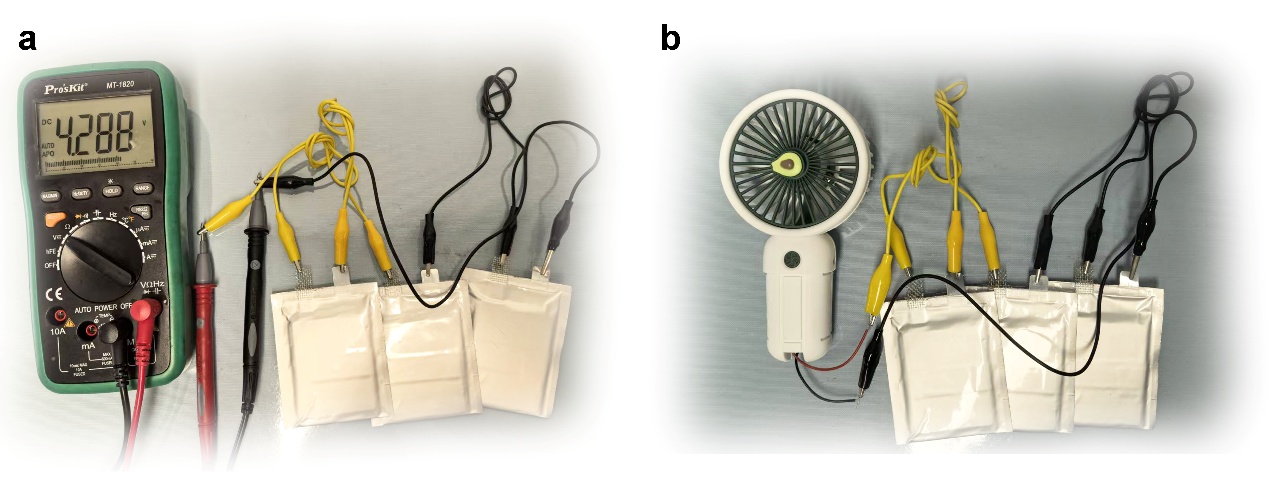


**Fig. S39** The digital photos of **a** the voltage of cells and **b** fan powering by three cells in series.

**Table S1** The cell size and atom count of Zn (slab) surface

|  | Cell size | | | Atom count |
| --- | --- | --- | --- | --- |
|  | a | b | c | Zn |
| Zn(002) | 13.133652 | 13.133652 | 35.207234 | 48 |
| Zn(100) | 10.506922 | 15.621702 | 35.307902 | 48 |
| Zn(101) | 11.664476 | 10.506922 | 34.516592 | 48 |
| Zn(102) | 13.829595 | 10.506922 | 33.997151 | 48 |

**Table S2** The mass of each component in pouch cell

| Cell components | Size | Mass |
| --- | --- | --- |
| Zn anode | 4.8 cm × 5.6 cm | 0.86 g |
| Cathode current | 4.8 cm × 5.6 cm | 0.42 g |
| Cathode | 4.8 cm × 5.6 cm | 0.85 g |
| Separator | 5.0 cm × 5.8 cm | 0.20 g |
| Electrolyte | 2 ml | 2.41 g |
| Al-plastic film | 8 cm × 12 cm | 1.10 g |
